# Chemokine GPCR Signaling Inhibits β-Catenin during Zebrafish Axis Formation

**DOI:** 10.1371/journal.pbio.1001403

**Published:** 2012-10-09

**Authors:** Shu-Yu Wu, Jimann Shin, Diane S. Sepich, Lilianna Solnica-Krezel

**Affiliations:** 1 Department of Biological Sciences, Vanderbilt University, Nashville, Tennessee, United States of America; 2 Department of Developmental Biology, Washington University School of Medicine, St. Louis, Missouri, United States of America; University of Pennsylvania School of Medicine, United States of America

## Abstract

Embryonic axis formation in vertebrates is initiated by the establishment of the dorsal Nieuwkoop blastula organizer, marked by the nuclear accumulation of maternal β-catenin, a transcriptional effector of canonical Wnt signaling. Known regulators of axis specification include the canonical Wnt pathway components that positively or negatively affect β-catenin. An involvement of G-protein coupled receptors (GPCRs) was hypothesized from experiments implicating G proteins and intracellular calcium in axis formation, but such GPCRs have not been identified. Mobilization of intracellular Ca^2+^ stores generates Ca^2+^ transients in the superficial blastomeres of zebrafish blastulae when the nuclear accumulation of maternal β-catenin marks the formation of the Nieuwkoop organizer. Moreover, intracellular Ca^2+^ downstream of non-canonical Wnt ligands was proposed to inhibit β-catenin and axis formation, but mechanisms remain unclear. Here we report a novel function of Ccr7 GPCR and its chemokine ligand Ccl19.1, previously implicated in chemotaxis and other responses of dendritic cells in mammals, as negative regulators of β-catenin and axis formation in zebrafish. We show that interference with the maternally and ubiquitously expressed zebrafish Ccr7 or Ccl19.1 expands the blastula organizer and the dorsoanterior tissues at the expense of the ventroposterior ones. Conversely, Ccr7 or Ccl19.1 overexpression limits axis formation. Epistatic analyses demonstrate that Ccr7 acts downstream of Ccl19.1 ligand and upstream of β-catenin transcriptional targets. Moreover, Ccl19/Ccr7 signaling reduces the level and nuclear accumulation of maternal β-catenin and its axis-inducing activity and can also inhibit the Gsk3β -insensitive form of β-catenin. Mutational and pharmacologic experiments reveal that Ccr7 functions during axis formation as a GPCR to inhibit β-catenin, likely by promoting Ca^2+^ transients throughout the blastula. Our study delineates a novel negative, Gsk3β-independent control mechanism of β-catenin and implicates Ccr7 as a long-hypothesized GPCR regulating vertebrate axis formation.

## Abstract 2

We identify a novel mechanism of β-catenin inhibition involving, chemokine-GPCR signaling, which limits axis formation during zebrafish embryogenesis.

## Author Summary

The arrangement of dorsoventral (back to belly) and anteroposterior (head to tail) embryonic structures is specified early during animal embryogenesis. In vertebrates, this process of axes formation is initiated by β-catenin, a transcriptional effector of Wnt signaling, which marks the prospective dorsal side. Thus far, all known regulators of axis specification that affect β-catenin include components of the Wnt pathway. G protein-coupled receptors (GPCRs), as well as Ca^2+^ signaling, have also been hypothesized to inhibit β-catenin and axis formation, however the identity of such GPCRs has remained unclear. Here, we identify two novel regulators of axis formation: the zebrafish Ccr7 chemokine GPCR and one of its ligands, Ccl19.1, both of which are uniformly expressed in the early zebrafish embryo. We show that reducing the expression of Ccr7 or Ccl19.1 increases β-catenin levels and also the dorsoanterior tissues at the expense of the ventroposterior ones. Conversely, an excess of Ccr7 or Ccl19.1 lowers β-catenin levels and limits axis formation. Further experiments show that Ccr7 acts upstream of β-catenin transcriptional targets. Our study delineates a novel mechanism for the negative control of β-catenin, whereby Ccr7 increases β-catenin degradation in a Gsk3b-independent manner, likely by promoting Ca^2+^ signaling throughout the embryo. In a new model of axis specification we propose that Ccr7 acts as a long-hypothesized GPCR that provides a global inhibition of β-catenin to ensure the precise formation of embryonic axes.

## Introduction

Early in vertebrate development, a cascade of inductive events patterns the dorsoventral (DV) and anteroposterior (AP) embryonic axes through the establishment of the embryonic organizing centers (for reviews, see [1]–[3]). First discovered in amphibians and subsequently in other vertebrates, the dorsovegetal blastula organizer, named the Nieuwkoop center, initiates embryonic axis formation and later induces the dorsal gastrula, or Spemann-Mangold organizer (SMO), which secretes factors antagonizing Bone morphogenetic protein (BMP) signaling (reviewed by [4]).

In frog and zebrafish, canonical Wnt signaling is critically involved in the formation and function of both dorsal signaling centers in two sequential phases, maternal and zygotic [3],[5]. The establishment of the Nieuwkoop center soon after fertilization is manifest by the nuclear accumulation of maternal β-catenin, a key transcriptional effector of the canonical Wnt pathway. At the onset of zygotic transcription, maternal β-catenin activates genes encoding transcription factors and secreted proteins that pattern embryonic axis. The maternal Wnt/β-catenin pathway has been implicated in axis determination by extensive evidence from gene perturbation studies, including zebrafish mutants (see [6] and references therein). Notably, maternal-effect zebrafish *ichabod* (*ich*) mutants, generated by females homozygous for a hypomorphic mutation in *β-catenin-2* locus, fail to establish the Nieuwkoop center and SMO. Consequently, *ich* mutants lack dorsoanterior and exhibit excess ventroposterior, embryonic structures [7].

To ensure the correct establishment of the embryonic organizers and polarity, the nuclear localization of β-catenin is under tight control. Interestingly, almost all known regulators of SMO formation are components of the Wnt/β-catenin pathway that directly or indirectly inhibit β-catenin, including Axin, Gsk3β, Naked1/2, and SCFβ-TrCP complex (see reviews in [8],[9]). For example, overexpression of Gsk3β in zebrafish leads to decreased expression of the SMO genes [10], whereas depleting maternal Naked1/2 elevates their expression [11]. On the other hand, genes such as *dickopff* (*dkk1*) and *frizzled-related protein* (*frzb*), encoding secreted Wnt inhibitors, are expressed in the SMO to restrict the ventralizing and posteriorizing Wnt8 activities [12],[13]. Collectively, limiting the activity of the Nieuwkoop center and SMO is indispensable for normal axis formation and entails both negative and positive modulation of the Wnt/β-catenin pathway, acting at the early and late blastula stages, respectively [14]–[16].

Several reports suggest intracellular Ca^2+^ as a negative regulator of axis specification through its antagonism of Wnt/β-catenin pathway (see reviews in [17],[18]). Although the molecular mechanisms regulating intracellular Ca^2+^ in early embryos remain elusive, mobilization of intracellular Ca^2+^ stores by activation of inositol trisphosphate (IP_3_) receptors in endoplasmic reticulum has been proposed to generate Ca^2+^ transients in the superficial blastomeres of zebrafish blastulae [19],[20]. In addition, β-catenin-independent or non-canonical Wnt signaling was proposed to counteract the canonical Wnt/β-catenin pathway during axis specification by promoting Ca^2+^ signaling [21],[22]. In support, overexpression of Wnt5 can antagonize β-catenin activity, resulting in ventralization [23]. However, it remains unclear whether Wnt5 is required for axis formation. Whereas according to some reports zebrafish *pipetail*/*wnt5b* mutants exhibit expansion of dorsal tissues [24], others reported normal axial patterning in embryos lacking *pipetail*/*wnt5b* [25]. Consequently, some current models of axis formation do not feature non-canonical Wnts or intracellular Ca^2+^ signaling [2]. Moreover, the mechanisms via which Ca^2+^ inhibits β-catenin in early embryos are not understood.

GPCRs have also been hypothesized to function as upstream regulators of Ca^2+^ mobilization during axis formation, based on the potential involvement of heterotrimeric G proteins in intracellular Ca^2+^ modulation in the zebrafish embryo [26],[27]. GPCRs constitute a large family of seven transmembrane receptors whose primary function is to transduce extracellular signals into cells. Upon ligand binding, GPCRs activate heterotrimeric G proteins, which modulate the propagation of various second messengers and/or the activity of ion channels. These receptors play prominent roles in sensory organs and the central nervous and immune systems in adults [28], as well as in cancer metastasis [29]. However, recent evidence implicates GPCRs, particularly the subgroup of chemokine receptors, in embryogenesis [30]. One of the chemokine signaling axes, the CXCL12/SDF-1-CXCR4/7, is involved in guiding movements of various cell populations, such as primordial germ cells [31],[32] or nascent endoderm [33],[34]. Also, Apelin and its cognate GPCR, Agtrl1b, control the migration and fate of cardiac precursors during zebrafish gastrulation [35],[36]. However, GPCRs involved in vertebrate axis formation have not yet been identified.

Here we report on a novel role for Ccr7, a chemokine GPCR known for its ability to control chemotaxis and other properties of lymphocytes [37], in regulating embryonic axis determination in zebrafish. Using loss- and gain-of-function approaches, we find that maternally and ubiquitously expressed Ccr7 and its ligand Ccl19.1 limit the formation of the Nieuwkoop and SMO organizers. Further epistasis and molecular analyses indicate that Ccr7 acts as a GPCR to negatively regulate the level and nuclear localization of maternal β-catenin by a Gsk3β-independent and Ca^2+^-dependent mechanism. We propose that Ccr7 GPCR signaling downstream of Ccl19.1 chemokine inhibits β-catenin in a Gsk3β-independent fashion, likely by promoting Ca^2+^ signaling throughout the blastula, and consequently limits the formation of the dorsal organizers and the embryonic axis.

## Results

### Ccr7 Activity Limits the Nieuwkoop Center and Axis Formation

**Figure 1:**
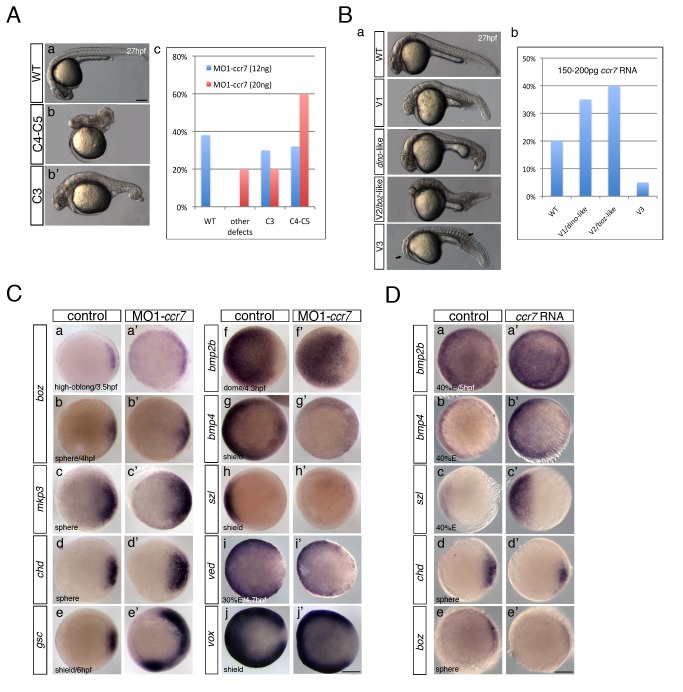
Ccr7 is required for proper AP and DV embryo patterning. (A) The spectrum of dorsalized phenotypes in embryos injected with MO1-*ccr7* at 27 hpf, ranging from highly dorsalized (C4–5; b) with truncated tails and trunks to C3 with tail deficiencies (b′) (total *n* = 190, seven independent experiments for 12 ng MO1-*ccr7* and *n* = 35, one experiment for 20 ng MO1-*ccr7*). The frequency of each phenotypic category is indicated in the right panel (c). The scale bars represent 200 µm in all figures. (B) Ccr7 overexpression (150–200 pg RNA) caused a spectrum of ventralized phenotypes, ranging from V3 to V1 (arrows show anterior and notochord deficiencies) to WT-like (a). The frequency of each phenotypic category is indicated in the right panel (b; *n* = 96, two experiments). (C) Expression of dorsal/ventral markers in Ccr7 morphants compared to control embryos revealed by WISH. (a–e′) Expression of dorsal genes was upregulated or expanded: at high-oblong stage (3.3–3.7 hpf), *boz*, *n* = 8/8; at sphere stage (4 hpf), *boz*, *n* = 16/28; *mkp3*, *n* = 9/12; *chd*, *n* = 11/15; at shield stage (6 hpf), *gsc*, *n* = 18/37. (f–j′) Expression of ventral genes was reduced: at dome (4.3 hpf) stage, *bmp2b*, *n* = 19/24; at 30% epiboly stage (4.7 hpf), *ved*, *n* = 12/14; at shield stage, *bmp4*, *n* = 9/10; *szl*, *n* = 11/12; *vox*, *n* = 16/22. Animal views, dorsal to the right. (D) Expression of dorsal/ventral markers in Ccr7-overexpressing embryos revealed by WISH. Expression of ventral markers was expanded (a–c′), while dorsal markers were decreased (d–e′): at sphere stage, *boz*, *n* = 8/8; *chd*, *n* = 10/13; at 40% epiboly stage (5 hpf), *bmp2b*, *n* = 12/12; *bmp4*, *n* = 14/14; *szl*, *n* = 13/13. Animal views, dorsal to the right.

During a large-scale characterization of the expression patterns of zebrafish chemokine GPCRs (SW and LSK, unpublished), we identified the *ccr7* ortholog [Chemokine (C-C motif) receptor 7] (Figure S1A; [38]) among GPCRs expressed during gastrulation. WISH (whole mount in situ hybridization) experiments revealed that *ccr7* RNA was expressed maternally and uniformly during early cleavage stages, becoming asymmetrically distributed by 4.5 hpf and dorsally enriched by early gastrulation (Figure S1B). To explore the role of Ccr7 in embryonic development, we interfered with its activity by injecting into early zygotes antisense morpholino oligonucleotides (MO1-*ccr7*) that effectively blocked *ccr7* translation by targeting 5′-UTR sequences, as revealed by the *ccr7*-*5′UTR-EGFP* reporter (Figure S1D). At 11 hpf (Figure S1C) and 27 hpf, the embryos injected with MO1-*ccr7* displayed a range of dorsalized phenotypes [39], including trunk and tail truncations (Class C4–C5; Figure 1Ab; all experimental numbers are provided in figure legends) or tail and ventral tail fin deficiencies (Class C3; Figure 1Ab′). These phenotypes were observed with variable penetrance and expressivity (Figure 1Ac), likely due to incomplete interference with the translation of maternal *ccr7* RNA and presence of maternal Ccr7 protein that could not be affected by the MO1-*ccr7*. Conversely, injections of synthetic *ccr7* RNA produced a spectrum of phenotypes, similar to ventralized zebrafish mutants (Figure 1B) [40]. Most exhibited minor (Class V1) or stronger axial defects (V2/*boz*-like; with reduced notochord, frequent cyclopia, but retaining some head structures), whereas in more severe cases (V3), the anterior head structures and notochord were completely absent (Figure 1Ba–b), similar to the phenotypes reported for strong *boz* [41] and *boz;sqt* double mutants [42],[43] or embryos overexpressing BMP ligands [40].

To characterize further the consequences of loss and gain of Ccr7 function on embryonic patterning, we examined the expression of a suite of region-specific markers (Figures 1C,D and S1G,H). Compared to the control blastula at 4 hpf, *ccr7* morphants showed expanded expression domains of the early dorsal markers, including three direct targets of maternal β-catenin, *boz*/*dharma* (Figure 1Ca–b′), *mkp3/dusp6* (Figure 1Cc–c′), and *squint* (*sqt*)/*ndr1* (Figure S1Ga–a′) [42],[44]. *Chordin* (*chd*), encoding a BMP antagonist, was also upregulated in *ccr7* morphants (Figure 1Cd–d′). Consistently, at early gastrulation (6 hpf), the expression domains of the SMO gene, *gsc* (Figure 1Ce–e′), and another β-catenin-dependent dorsal gene, *hhex*, were also significantly broadened (Figure S1Gb–b′). Conversely, the ventrally restricted genes, *bmp2b* and *bmp4*, were downregulated and/or exhibited reduced expression domains (Figure 1Cf–g′). Likewise, the ventrally expressed and BMP-dependent gene, *sizzled* (*szl*), was strongly downregulated in *ccr7* morphants (Figure 1Ch–h′), while the expression of *vox*, *vent*, and *ved*, all members of the *vox/vent* gene family, were more ventrally restricted (Figure 1Ci–j′ and Figure S1Gc–c′). Using qRT-PCR to measure changes in the expression level of selected genes in *ccr7* morphants, we observed that soon after the initiation of zygotic transcription [45], expression of *boz* and another early dorsal gene, *fgf3*, was slightly but significantly upregulated (3.3 hpf; 1.2 to 1.6 relative fold expression), whereas *bmp2b* expression was mildly decreased. Soon thereafter (4 hpf), the expression of *mkp3* was highly increased, while *boz* expression remained slightly elevated (Figure S1Gd).

To test whether the dorsalized phenotype of *ccr7* morphants resulted from specific interference of MO1 with *ccr7* translation, we co-injected MO1-*ccr7* and a synthetic *ccr7* RNA lacking MO1-targeting sequences. We observed that both the penetrance and expressivity of the morphological (not shown) and gene expression aspects of the dorsalized phenotype of *ccr7* morphants were suppressed, as shown by *szl* expression (Figure S1E). In addition, injections of a control MO harboring five mismatches in MO1-*ccr7* target sequence had no effect on embryo morphology or expression of dorso-ventral markers at blastula stages (Figure S1F). All together, these data indicated that *ccr7*-deficient embryos exhibited enlarged Nieuwkoop center and SMO and subsequently expansion of dorsoanterior at the expense of ventroposterior cell fates.

Consistent with the above model, embryos injected with *ccr7* RNA exhibited ventralized characteristics at 27 hpf (Figure 1Ba,b) and, at earlier stages, expanded expression domains of the ventral and posterior genes *bmp2b*, *bmp4*, *szl*, *ved*, and *vox* (Figure 1Da–c′, Figure S1Hc–e′,h–h′) and reduced expression domains of *boz*, *mkp3*, and *chd* (Figure 1Dd–e′, Figure S1Ha–b′,f–g′). Based on these results, we propose that maternally provided *ccr7* is required during zebrafish axis formation to limit the Nieuwkoop center and SMO and subsequently development of dorsoanterior embryonic structures.

### Ccr7 Limits Axis Formation by Negatively Regulating β-Catenin

Next, we set out to delineate the position and mechanism of Ccr7 action within the axis formation genetic hierarchy. Owing to its maternal expression and effects on the early Nieuwkoop center markers like *boz* or *sqt* (Figures 1C,D and S1H), which are direct targets of β-catenin, we hypothesized that Ccr7 acts by regulating the maternally provided β-catenin. We first examined its relationship to the *boz* gene, which is essential for SMO formation [41] and whose overexpression hyper-dorsalizes embryos by inducing ectopic expression of SMO genes, such as *gsc* (Figure S2A and S2B; [46],[47]). However, co-injection of *ccr7* with *boz* RNA could not suppress the hyper-dorsalized phenotypes resulting from Boz overexpression (Figures S2A and S2B). The results of this epistasis experiment support the notion that Ccr7 limits axis formation by acting upstream of *boz* and thus likely by regulating β-catenin.

**Figure 2:**
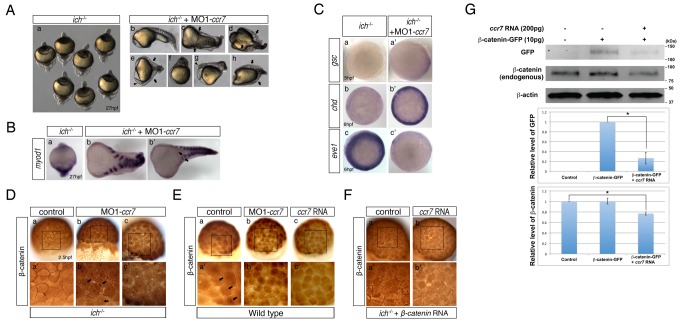
Depletion of Ccr7 activity partially rescues axis formation in *ichabod* mutants. (A) Penetrance of strongly ventralized phenotypes displayed by maternal β-catenin-2, *ich*, mutant embryos (a, 92%, *n* = 110) was reduced by injection of MO1-*ccr7* (b–h, 10 ng; 40%, *n* = 98, five experiments). Arrows indicate partial axes and arrowheads indicate rudimentary head structures. (B) *myod1* expression in uninjected (a) and MO1-*ccr7-*injected *ich* embryos (b,b′) revealed somitic tissue. Arrows indicate partial double axes. (C) In contrast to uninjected *ich* mutants (a,b,c), in MO1-*ccr7*-injected *ich* embryos, the organizer genes *gsc* (a′, *n* = 11/12) and *chd* (b′, *n* = 8/8) were expressed; while expression of the ventral gene, *eve1,* was significantly reduced (c′, *n* = 30/40). Animal views. (D) Lack of β-catenin nuclear accumulation, detected by immunostaining at 256-cell stage, in *ich* mutants (a), was suppressed in MO1-*ccr7*-injected *ich* embryos (b,c; *n* = 9/10, two experiments). (a′–c′) Higher magnification of boxed areas in a–c. (E) Dorsal domain of nuclear accumulation of β-catenin, detected by immunostaining at 256-cell stage in WT embryos (a), was expanded in *ccr7* morphants (b; *n* = 6/13), while it was diminished in Ccr7 overexpressing embryos (c; *n* = 8/11). (a′–c′) Higher magnification of boxed areas in a–c. (F) Ectopic β-catenin nuclear accumulation, detected by immunostaining at 256–512-cell stage, in *ich* mutants injected with *β-catenin* RNA (a; 25 pg, *n* = 9/10), was suppressed by co-injecting *ccr7* RNA (b; 150 pg, *n* = 7/10). (a′–b′) Higher magnification of boxed areas in a–b. (G) Ccr7 gain-of-function decreased both levels of endogenous β-catenin and ectopic β-catenin-GFP. Western blotting of β-catenin and GFP protein from uninjected control, *β-catenin-GFP* RNA (10 pg) injected, or *β-catenin-GFP* RNA (10 pg)/*ccr7* RNA (200 pg) co-injected embryos (all at 3–3.3 hpf). Graphs below show the relative protein level (signal intensity) quantified from three separate immunoblots. * *p*<0.05.

To test this hypothesis more directly, we utilized the maternal-effect hypomorphic regulatory *ich^p1^* mutation, which reduces expression of the *β-catenin-2* locus [7],[16]. Accordingly, almost all of the progeny of *ich^p1/p1^* females, called thereafter *ich* mutants, exhibited the strongest ventralized or radialized phenotypes, characterized by the lack of head, severely reduced trunk, and enlarged tail (Figure 2Aa). Strikingly, *ich* mutants injected with MO1-*ccr7* at the 1-cell stage displayed a phenotypic spectrum, with over half of the embryos exhibiting some trunk tissues, including somites (Figure 2Ab–e, arrows) and some showing rudimentary head structures (Figure 2Ae,g, arrowheads). Intriguingly, a fraction of these MO1-*ccr7*-injected *ich* mutants formed partial double axes with a common posterior tail (20%–25%; Figure 2Ac,d,e,h, arrows), as also revealed by expression of *myod1*, a somitic marker (Figure 2B).

Gene expression analyses at early gastrulation indicated that, in a striking contrast to control *ich* mutant embryos (Figure 2Ca–b), the SMO genes *chd* and *gsc* were expressed more broadly or around the embryonic circumference of MO1-*ccr7*-injected *ich* mutants (Figure 2Ca′–b′). Conversely, expression of the ventrolateral marker *eve1* was strongly reduced in MO1-*ccr7*-injected *ich* mutant gastrulae, which are characterized by expanded expression of this gene (Figure 2Cc–c′) [7]. In conclusion, this attenuation of *ich* phenotypic severity by reducing *ccr7* function is consistent with the notion that Ccr7 plays a negative role in dorsal axis specification by directly or indirectly regulating β-catenin.

Next, we asked whether the partial suppression of the *ich* mutant phenotype by Ccr7 depletion was due to an influence on β-catenin expression. β-catenin is detected in nuclei on the prospective dorsal side of 128–1,000 cell wild-type (WT) blastulae (Figure 2Ea,a′) [48],[49]. In contrast, *ich* mutants have very reduced levels of maternal β-catenin, which is undetectable in the nuclei (Figure 2Da–a′) [7]. Strikingly, most cells in *ich* mutant blastulae injected with MO1-*ccr7* showed nuclear accumulation of β-catenin (Figure 2Db–c′). Further, compared to control WT embryos, the dorsal domain of nuclear localized β-catenin was expanded in WT embryos injected with MO1-*ccr7*, while it was reduced in WT blastulae overexpressing Ccr7 (Figure 2E). Moreover, Western blot analyses revealed reduced levels of endogenous maternal β-catenin and β-catenin-GFP in embryos co-injected with *β-catenin-GFP* and *ccr7* RNA (Figure 2G; 3–3.3 hpf) and also after the onset of the zygotic transcription (Figure S2E; 4 hpf). Finally, *ich* mutants injected with synthetic *β-catenin* RNA showed increased level and nuclear accumulation of β-catenin that was eliminated by co-injection of *ccr7* RNA (Figures 2F and S2E). Based on the above results, we conclude that Ccr7 negatively regulates the level and nuclear localization of β-catenin.

### Ccr7 Inhibits Axis-Inducing Activity of β-Catenin in a Gsk3β-Independent Manner

**Figure 3:**
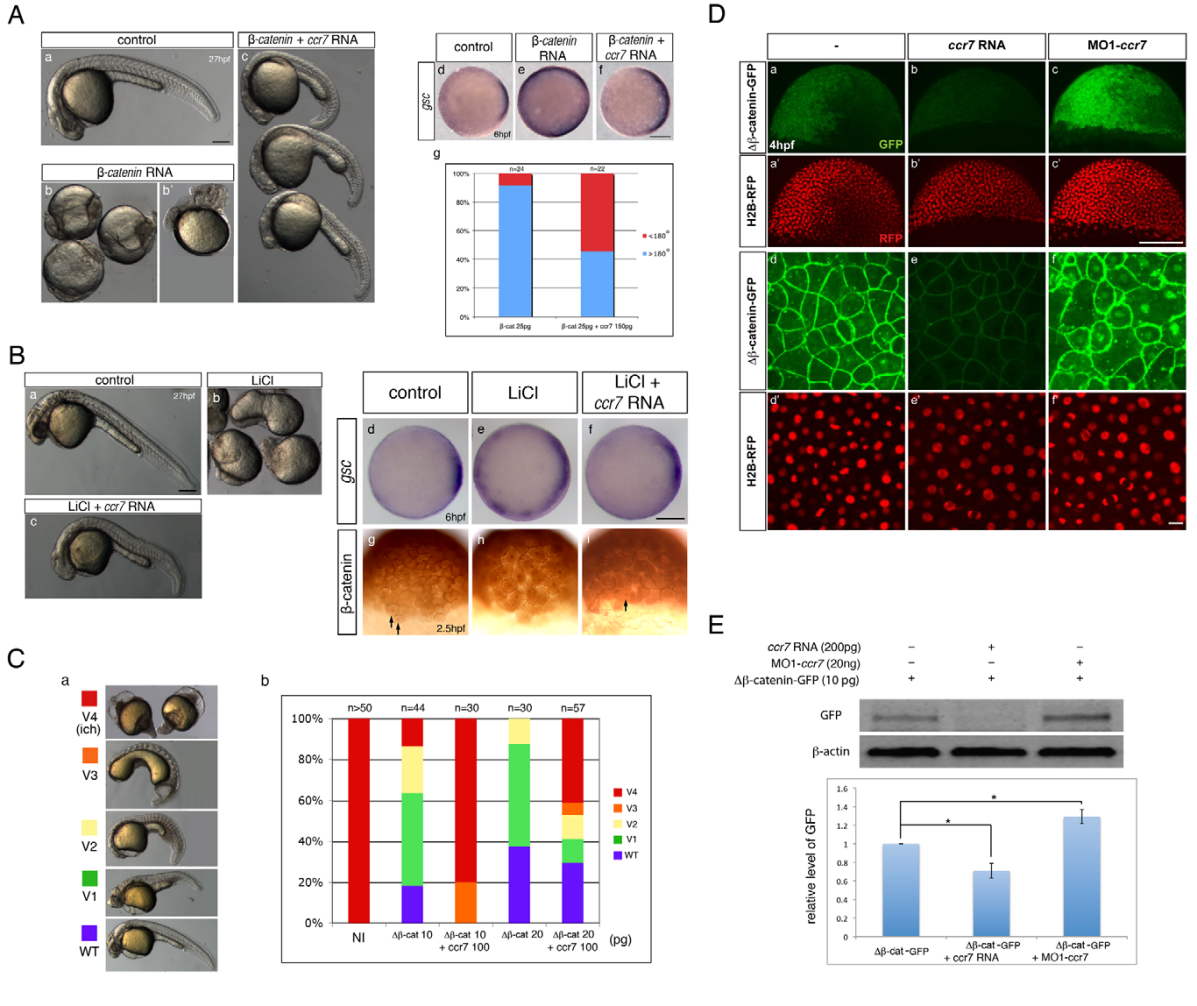
Ccr7 inhibits β-catenin activity via a Gsk3β-indepenedent mechanism. (A) Hyper-dorsalized phenotypes caused by β-catenin overexpression (b,b′, 25 pg, *n* = 22/25), compared to control WT embryos (a), were suppressed by Ccr7 overexpression (c; 150 pg, *n* = 8/12). (d–f) Expansion of *gsc* expression domain in β-catenin overexpressing embryos (e), relative to control WT embryos (d), was suppressed by co-injection of *ccr7* RNA (f). Animal views, dorsal to the right. (g) Frequency of embryos with *gsc* expression domain encompassing more (>180°) or less (<180°) than half of the embryo equator. (B) (a–c) LiCl-treated embryos (b; *n* = 16/20) show dorsalized phenotypes at 30 hpf compared to control embryos (a). LiCl-dependent dorsalization was suppressed by injection of *ccr7* RNA (c; *n* = 8/20, two experiments). (d–f) *gsc* expression at shield stage (6 hpf) in control (d), LiCl-treated (e; *n* = 13/14), and LiCl-treated and *ccr7* RNA-injected embryos (f; *n* = 9/12). Animal views, dorsal to the right. (g–i) β-catenin immunostaining at 256-cell stage in control (g), LiCl-treated (h, *n* = 9/10), and LiCl-treated embryos overexpressing Ccr7 (i; *n* = 9/11). Arrows point to a few β-catenin-positive nuclei in control embryos (g) and LiCl-treated embryos overexpressing Ccr7 (i). (C) Ccr7 antagonizes the ability of ΔNβ-catenin to rescue the ventralized *ich* mutant phenotype. (a) V1–V4 phenotypic classes, with V4 corresponding to the strongest *ich* phenotype. (b) Frequencies of the V1–V4 phenotypic classes of *ich* mutants injected with synthetic *ΔNβ-catenin* RNA alone or co-injected with *ccr7* RNA. Injected amounts of RNAs in pg are shown below the graph, and the number of embryos in each group above each bar. (D) (a–c) Co-injections of *ΔNβ-catenin-gfp* RNA and MO1-*ccr7* or *ccr7* RNA showed that Ccr7 can downregulate β-catenin, shown at higher-magnification (d–f). Compared to control (a, d), *ccr7* RNA overexpressing blastulae showed strongly decreased (b, e), while MO1-*ccr7* injected blastulae showed increased, ΔNβ-catenin-GFP signal (c, f). *H2B-RFP* RNA was injected as nuclear background control (a′–c′ and higher magnification in d′–f′). (E) Western blot analysis of co-injection of *ΔNβ-catenin-gfp* RNA and *ccr7* RNA or MO1-*ccr7*. Quantification of the relative protein level (signal intensity) from three independent immunoblots (bottom panel). * *p*<0.05.

To define the underlying mechanism by which Ccr7 inhibits β-catenin, we tested the ability of Ccr7 to antagonize WT and mutant β-catenin in co-expression experiments. Consistent with previous reports [50], injection of synthetic *β-catenin* RNA into WT zygotes resulted in strong embryo dorsalization (Figure 3Ab–b′), correlated with increased expression of the SMO marker *gsc* (Figure 3Ae). In support of the notion that Ccr7 inhibits β-catenin, the dorsalized phenotypes of β-catenin overexpressing embryos were suppressed by co-injecting *ccr7* RNA (Figure 3Ac,f,g). Similarly, Ccr7 overexpression suppressed the rescuing effect of *β-catenin* RNA on *ich* mutant phenotype (Figure S2C).

One of the key negative regulators of β-catenin is Gsk3β, which phosphorylates β-catenin and targets it to proteasome-mediated degradation [51],[52]. To investigate if the negative control of β-catenin by Ccr7 is dependent on Gsk3β activity, we employed two different approaches. First, we treated zebrafish blastulae with LiCl, a well-known Gsk3β inhibitor, which results in stabilization of β-catenin [53], dorsalized embryo morphology (Figure 3Ba–b), and enlarged *gsc* expression domain (Figure 3Bd–e), consistent with previous reports [54]. Interestingly, both the dorsalized morphology and the expansion of *gsc* expression domain caused by LiCl treatment were significantly suppressed in embryos overexpressing Ccr7 (Figure 3Bc and Bf). Moreover, the expansion of the domain of nuclear β-catenin localization in LiCl-treated, compared to the control, WT blastulae was suppressed by Ccr7 overexpression (Figure 3Bg–i).

Second, we utilized an N-terminally truncated mutant form of β-catenin (ΔNβ-catenin), which cannot be targeted by Gsk3β for degradation [52]. Injection of synthetic *ΔNβ-catenin* RNA effectively suppressed the ventralized phenotypes of *ich* mutants in a dose-dependent manner, producing mutants with largely WT appearance or only mild axial defects (classes V1–V2; Figure 3C). However, when the synthetic *ΔNβ-catenin* and *ccr7* RNAs were co-injected into *ich* zygotes, the resulting embryos represented the more severe section of the phenotypic *ich* mutant spectrum (V3–V4; Figure 3C). Conversely, the ability of ΔNβ-catenin-GFP to dorsalize WT embryos was enhanced by simultaneously depleting Ccr7 (Figure S2D). In addition, the level and nuclear localization of ΔNβ-catenin-GFP was significantly reduced in WT blastulae co-injected with *ccr7* RNA and increased when *ΔNβ-catenin* RNA was co-injected with MO1-*ccr7*, as seen by in vivo imaging (Figure 3D) or Western blotting (Figure 3E).

To explore further the mechanism via which Ccr7 downregulates β-catenin levels, we employed Lactacystin, a known inhibitor of proteasome activity (see Materials and Methods). As shown in Figure S3, Lactacystin treatment of early embryos expressing β-catenin-GFP fusion protein and Ccr7 or Gsk3β partially suppressed the reduction of β-catenin-GFP levels in embryos overexpressing Ccr7 as well as in embryos overexpressing Gsk3β. Collectively, these data support the conclusion that Ccr7 negatively regulates β-catenin levels through a Gsk3β-independent mechanism, in part via proteasome-dependent degradation.

### Ccr7 Functions as a GPCR to Limit Axis Formation

**Figure 4:**
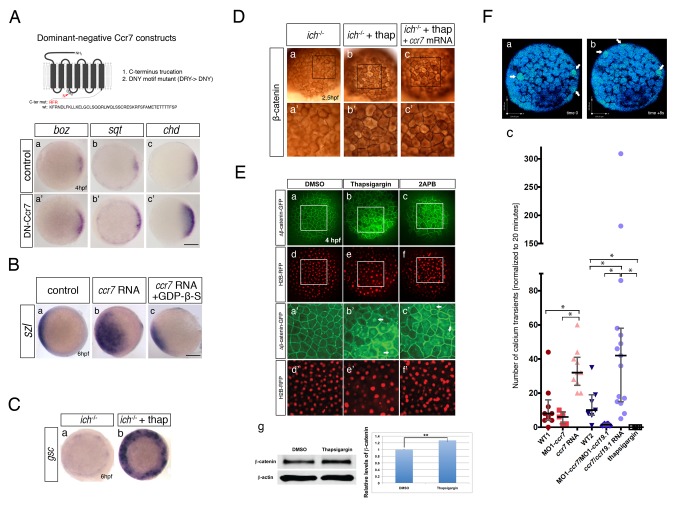
Ccr7 functions as a GPCR and promotes calcium signaling. (A) Misexpression of two dominant-negative Ccr7 mutant forms promotes organizer gene expression. Top panel, a schema of the mutant forms employed: DN1, C-terminal truncation, and DN2, DRY→DNY mutant. Bottom panel shows effects of injecting 100 pg *DN2-ccr7* RNA on *boz* (a′, *n* = 14/18) and *sqt* (b′, *n* = 11/15), and of *DN1-ccr7* RNA on *chd* (c′, *n* = 13/20) expression compared to uninjected control embryos (a–c) at sphere stage, 4 hpf. Animal views, dorsal to the right. (B) Expression of *szl* in control embryos (a, *n* = 8/8) was expanded in *ccr7* RNA injected WT embryos (b, 150 pg, *n* = 14/14), and suppressed by co-injection of GDP-β-S (∼300 nM) at shield stage (c, *n* = 12/15, two experiments). Animal views, dorsal to the right. (C) *gsc* expression in control (a) and thapsigargin-treated *ich* mutants (b; 4 µM; *n* = 20/20) at shield stage, 6 hpf. Animal views, dorsal to the right. (D) β-catenin immunostaining in control (a, a′), thapsigargin-treated *ich* mutants (b, b′; 4 µM; *n* = 6/6), and thapsigargin-treated *ich* mutants injected with *ccr7* RNA (c, c′; *n* = 5/6). (a′–c′) Higher magnification images of the boxed areas in a–c. (E) Thapsigargin (b, b′) and 2-APB-treated WT embryos (c, c′) showed increased level and nuclear ΔNβ-catenin-GFP signal, compared to control embryos (a, a′). *H2B-RFP* RNA was injected as nuclear background control (d, d″, e, e′, c, c′). (d) Western blot for total β-catenin and actin in control (DMSO-treated) and thapsigargin-treated (4 µM) WT embryos at 4 hpf, with relative quantification to β-actin from three independent experiments in the right panel. (F) Effects of *ccr7* and thapsigargin on Ca^2+^ transients in superficial blastomeres. (a, b) Examples of Ca^2+^ transients revealed by in vivo ratiometric image analysis in a WT embryo at 512-cell stage. The arrows point out the Ca^2+^ transients at consecutive time points (still images). Note the rapid changes of Ca^2+^intensity (compare b to a, 8 s interval). (c) The number of Ca^2+^ transients normalized to 20 min in control embryos (WT1, *n* = 9, red; WT2, *n* = 7, blue), *ccr7* morphants (*n* = 5), Ccr7 overexpressing blastulae (*n* = 10), *ccr7/ccl19.1* double morphants (20 ng/2 ng; *n* = 8), and Ccr7/Ccl19.1 overexpressing (200 pg/200 pg RNA; *n* = 15), and thapsigargin-treated (*n* = 4) blastulae were quantified by in vivo time-lapse imaging with Calcium Green-1 at about 256-cell stage or 2.5 hpf (see Materials and Methods for detail). Red and blue data were collected over 10 and 20 min, respectively; * *p*<0.05, Student's *t* test, unequal variance.

Next we asked whether Ccr7 regulates β-catenin and axis formation by functioning as a bona fide GPCR. We first generated dominant-negative constructs of Ccr7, either lacking the C-terminus (aa 331–372) or mutating the DRY motif, previously shown to be indispensable for the mammalian Ccr7 to induce downstream signaling events, including Ca^2+^ mobilization [55]. In agreement with Ccr7 influencing axis formation as a GPCR, injection of synthetic RNAs encoding either dominant-negative form of Ccr7 increased expression of dorsal markers *boz*, *sqt*, and *chd* (Figure 4Aa–c′), phenocopying defects observed in Ccr7 morphants (Figure 1C). Second, we tested whether Ccr7, as expected from a chemokine GPCR, signals through G-protein coupling in the process of axis formation. Consistent with this notion, treating Ccr7 overexpressing embryos with GDP-β-S, a GPCR/heterotrimeric G protein activation inhibitor [56], suppressed the ventralizing effect of Ccr7, as revealed by *szl* expression (Figure 4B).

**Figure 5:**
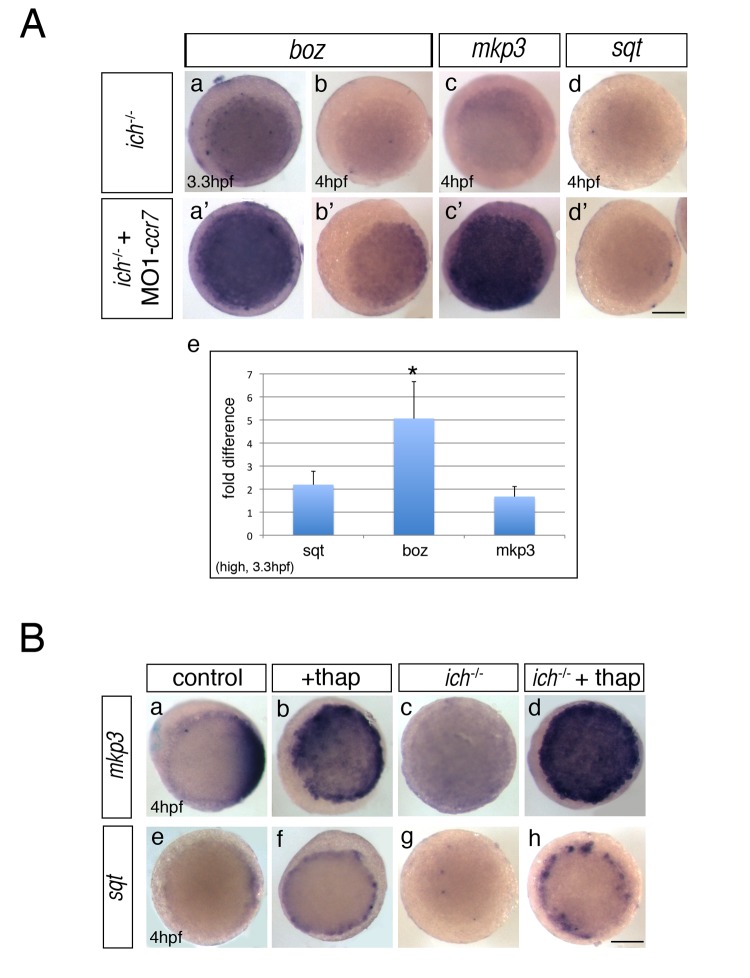
Inhibition of Ccr7 and intracellular calcium promotes expression of dorsal genes. (A) Expression of β-catenin downstream targets, *boz*, *mkp3*, and *sqt*, in *ich* mutants (a–d) and MO1-*ccr7*-injected *ich* mutants (a′–d′) during early blastula stages revealed by WISH. (a–a′) 3.3 hpf, *boz*, *n* = 11/14; (b–b′) *boz*, *n* = 6/11; and 4 hpf (c–c′) *mkp3*, *n* = 8/8; (d–d′) *sqt*, *n* = 8/8. Animal views, dorsal to the right. (e) Quantification of the relative expression levels of *sqt*, *boz*, and *mkp3* in *ich* mutants and MO1-*ccr7*-injected *ich* mutants by qRT-PCR; * *p*<0.05, Student's *t* test. (B) The effect of thapsigargin on the expression of *mkp3* and *sqt* in WT and *ich* embryos at sphere stage, 4 hpf. (a–d) *mkp3*: b, *n* = 9/9; d, *n* = 13/13. (e–h) *sqt*: f, *n* = 6/6; h, *n* = 8/8. Animal views.

We next sought to delineate the events downstream of Ccr7 that lead to β-catenin inhibition. Mammalian Ccr7 has been shown to mobilize intracellular Ca^2+^ as one of the downstream responses [57]. In addition, previous reports suggested Ca^2+^ as a negative regulator of zebrafish axis formation, acting possibly as an inhibitor of the Wnt/β-catenin pathway [17],[18]. Therefore, we tested whether Ccr7 inhibits β-catenin through the activation of Ca^2+^ signaling. We first used a known Ca^2+^ inhibitor, thapsigargin [58], to effectively block the intracellular Ca^2+^ fluxes (Figure 4F) [19],[20]. Compared to the untreated and strongly ventralized *ich* mutants, thapsigargin-treated *ich* embryos showed some axial characteristics, such as *myod1*-expressing somites (unpublished data). Also, expression of dorsal markers, including *gsc* (Figure 4C) and *mkp3*, *sqt* (Figure 5Bc–h), was strongly upregulated, surrounding the whole margin of the thapsigargin-treated *ich* embryos. These results suggest that suppression of intracellular Ca^2+^ transients can activate the axis induction gene hierarchy in *ich* mutants, phenocopying loss of *ccr7* function in these mutants (compare Figures 4C, 5B and 2C, 5A). Also reminiscent of *ccr7-*deficient *ich* mutants, increased level and nuclear localization of endogenous β-catenin was detected in thapsigargin-treated *ich* mutant blastulae (Figure 4Db–b′). Consistently, thapsigargin-treated WT embryos showed increased levels and nuclear localization of misexpressed ΔNβ-catenin (Figure 4Ea–e′) and increased levels of endogenous β-catenin, as revealed by western blotting (Figure 4Eg). Additionally, WT embryos treated with 2-APB, an antagonist of IP_3_ receptors regulating mobilization of intracellular Ca^2+^ in early zebrafish blastulae [19], also showed increased levels and nuclear accumulation of ΔNβ-catenin (Figure 4Ec–f′) and upregulated *mkp3* expression similar to thapsigargin-treated WT embryos (Figure S4C). Thus, Ccr7 could act upstream of IP_3_-mediated intracellular Ca^2+^ fluxes, consistent with IP_3_-mediated Ca^2+^ release from intracellular stores negatively regulating β-catenin [27]. It was noted that embryos injected with MO1-*ccr7* (Figure 3D) and treated with thapsigargin or 2APB (Figure 4E) also displayed heterogeneous cell sizes and shapes; these differences may reflect abnormalities in cell division and cell shape, consistent with known effects of Ca^2+^ on cytokinesis [59] and cell shape changes [60]. At 1 dpf embryos treated with thapsigargin or 2APB displayed a variety of defects that cannot be simply accounted by dorsalization, including shortened and malformed body axes and yolk cells incompletely covered by the blastoderm (Figure S4D). These abnormalities are likely due to the effects of inhibition of Ca^2+^ stores on epiboly [61],[62] and convergence and extension gastrulation movements [63], in addition to the DV patterning defects.

To test whether such a Ccr7/Ca^2+^ pathway operates in zebrafish, we directly monitored the dynamic Ca^2+^ release in vivo during early cleavage stages by ratiometric time-lapse imaging (Figures 4F, S4A and S4B; also see Movies S1–S3 and Materials and Methods for details). Aperiodic, localized Ca^2+^ transients occur starting at 32 to 1K cell stage (1.75–3 hpf) and are uniformly distributed in the superficial blastomeres of zebrafish blastulae [20],[56], concurrent with the nuclear localization of β-catenin on the prospective dorsal side [48]. Compared to the WT control, the frequency of Ca^2+^ transients was significantly higher in embryos overexpressing Ccr7 as well as in embryos co-expressing Ccr7 and its Ccl19.1 chemokine ligand (see below), correlating with a reduction of β-catenin in embryos overexpressing Ccr7 (Figures 2G, 2E, and 3D,E). Conversely, the frequency of Ca^2+^ transients was reduced with incomplete penetrance in *ccr7* (or *ccr7/ccl19.1*) morphants compared to un-injected controls. Finally, strong and consistent reduction of Ca^2+^ transients was observed in thapsigargin-treated WT embryos (Figures 4F, S4A and S4B), correlating with a marked upregulation of β-catenin levels upon thapsigargin treatment (Figures 4Eb–e′,g).

If excess Ccr7 negatively regulated β-catenin by mobilizing intracellular Ca^2+^, reducing intracellular Ca^2+^ levels should block this effect. Accordingly, thapsigargin-treated *ich* mutants overexpressing Ccr7 showed a broad nuclear localization of β-catenin (Figure 4Dc–c′), indicating that reducing intracellular Ca^2+^ is epistatic to *ccr7* gain-of-function. All together, these data support a model whereby Ccr7 GPCR signaling induces, as one of its downstream responses, Ca^2+^ signaling, which in turn negatively regulates the level and nuclear localization of β-catenin and consequently limits axis formation during the cleavage stages of development.

Unlike the complete rescue of *ich* phenotypes caused by misexpression of β-catenin or Boz (Figures 3C, S2C, and unpublished data) [7], *ich* mutants depleted of either *ccr7* function (Figure 2A and 2B) or intracellular Ca^2+^ did not develop complete axes, even though the nuclear-localized β-catenin (Figures 2D and 4D) and *gsc* gene expression (Figures 2C and 4C) were clearly observed at the blastula stage. These differences could indicate that the Ccr7/Ca^2+^ pathway influences the dorsal gene expression/axis formation in addition to its effects on β-catenin. Therefore, we examined the expression of three early β-catenin-dependent genes in MO1-*ccr7*-injected *ich* mutants, following the initiation of zygotic transcription by qRT-PCR and WISH. Interestingly, *boz* expression was initially uniformly activated in MO1-*ccr7*-injected *ich* mutants at 3.3 hpf (Figure 5Aa′,Ae),but was decreased by 4 hpf (Figure 5Ab′). In contrast, expression of *mkp3* showed a moderate increase at first (Figure 5Ae) but was then strongly upregulated, encompassing the entire blastoderm at 4 hpf (Figure 5Ac′). The gradual reduction of *boz* expression from 3.3 to 4 hpf contrasted the increasing expression of *mkp3* (Figure 5Aa′–c′) in *ich* mutants injected with MO1-*ccr7*, similar to the changes in expression of these two genes in WT embryos injected with MO1-*ccr7* (Figure S1Gd). These observations are consistent with previous reports that maintenance, but not initiation, of *boz* expression is dependent on FGF signaling and that overexpression of *mkp3*, encoding a feedback attenuator of the FGF pathway, limits *boz* expression [64].

Thapsigargin-treated WT embryos (Figure 5Bb) and MO1-*ccr7*-injected *ich* mutants (Figure 5A,B) showed comparable gene expression profiles at 4 hpf, including greatly expanded *mkp3* expression (Figure 5Bd), also observed in WT embryos treated with 2-APB (Figure S4Cc). On the other hand, the expression of another β-catenin target gene, *sqt*, was slightly increased in thapsigargin-treated WT and *ich* embryos (Figure 5Bf,h), and less so in MO1-*ccr7*-injected *ich* mutants (Figure 5Ad′), suggesting that MO1-*ccr7* may only partially block the Ca^2+^ release, consistent with the Ca^2+^ imaging data (Figure 4F). In summary, we observed that *mkp3*, encoding an FGF antagonist, became highly upregulated relative to other SMO markers between 3 and 4 hpf in both WT blastulae (Figures 5B and S1Gd) and *ich* mutants (Figure 5A). Because formation of a complete embryonic axis in *ich* mutants requires FGF signaling [64], the upregulation of *mkp3* could account for the formation of incomplete axes in *ich* mutants injected with MO1-*ccr7* and treated with thapsigargin.

### Ccl19.1 Signals through Ccr7 to Regulate Axis Formation

**Figure 6:**
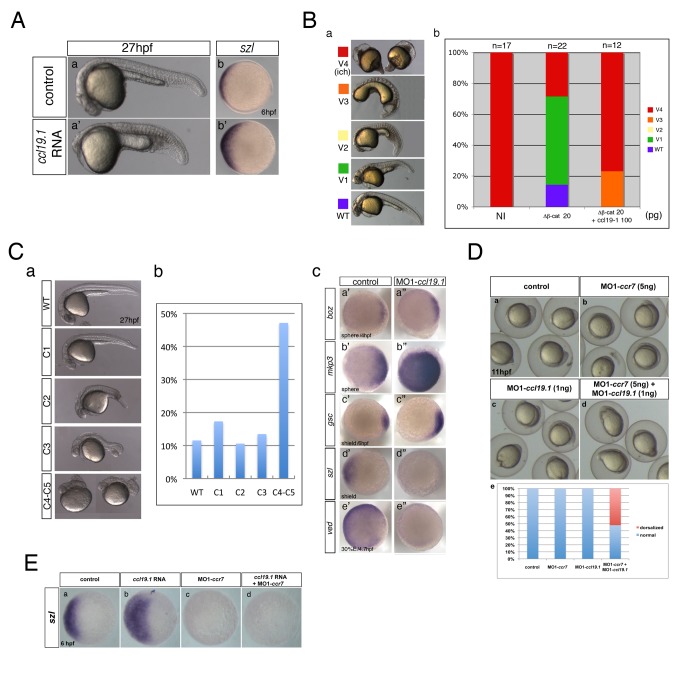
Ccl19.1 chemokine functions as a Ccr7 ligand in axis formation. (A) Injection of *ccl19.1* RNA (100–120 pg) resulted in ventralized embryo morphology at 27 hpf (a, a′; *n* = 30/45; lateral views with anterior to the left) and expansion of *szl* expression domain (b′) compared to control (b; *n* = 12/15). Shield stage, animal views with ventral to the left. (B) Ccl19.1 antagonizes the ability of ΔNβ-catenin to rescue the ventralized *ich* mutant phenotype. (a) V1–V4 phenotypic classes, with V4 corresponding to the strongest *ich* phenotype (also shown in Figure 3Ca). (b) Frequencies of the V1–V4 phenotypic classes of *ich* mutant embryos injected with synthetic *ΔNβ-catenin* RNA alone or co-injected with *ccl19.1* RNA. Injected amounts of RNAs in pg are shown below the graph, and the number of embryos in each group, above each bar. (C) (a) The spectrum of dorsalized phenotypes at 27 hpf in embryos injected with 4 ng MO1-*ccl19.1* classified into five categories, ranging from C4–C5 (the most severe class) to WT-like. (b) Frequency of each phenotypic category (*n* = 104, three experiments). (c) WISH analysis of dorsal/ventral markers in *ccr19.1* morphants compared to control blastulae. (a′–c″) Expression of dorsal genes was upregulated or expanded: sphere (4 hpf), *boz*, *n* = 22/31; *mkp3*, *n* = 11/11; shield (6 hpf), *gsc*, *n* = 9/10. (d′–e″) Expression domains of ventral genes were reduced: 30% epiboly (4.7 hpf), *ved*, *n* = 10/12; shield (6 hpf), *szl*, *n* = 11/12. Animal pole views, dorsal to the right. (D) Co-injection of MO1-*ccr7* and MO1-*ccl19.1* leads to dorsalization in a synergistic fashion. Injection of low doses of MO1-*ccr7* (b; *n* = 24) and MO1-*ccl19.1* (c; *n* = 26) alone did not cause dorsalized phenotypes, as observed for uninjected control embryos (a; 11 hpf, *n* = 36). (d) Embryos co-injected with the same doses of both MOs resulted in dorsalization (*n* = 12/23). (e) Frequency of dorsalized embryos in a–d. (E) *szl* expression was expanded in *ccl19.1* RNA-injected (b; 100 pg), compared to uninjected, control embryos (a) and was reduced in MO1-*ccr7*-injected (c; 12 ng) and *ccl19.1* RNA (100 pg) and MO1-*ccr7* (12 ng) co-injected embryos (d; two experiments). See text for details. Animal views of shield stage embryos, dorsal to the right.

To identify a ligand that regulates Ccr7 in the process of axis formation, we analyzed one of the zebrafish homologs of the known mammalian Ccr7 ligands, Ccl19.1 [65], which is expressed maternally and ubiquitously during early cleavage stages (unpublished data). Consistent with Ccl19.1 activating Ccr7 activity during axis formation, injection of synthetic *ccl19.1* RNA ventralized embryos (Figures 6A, S5A, and S5D), phenocopying Ccr7 overexpression (Figure 1B). In addition, Ccl19.1 overexpression counteracted the axis-inducing activity of β-catenin (Figure 6Bb), as Ccr7 overexpression did (Figure 3Cb). To test if Ccl19.1 is required for axis formation, we injected MO1-*ccl19.1* to interfere with its translation. We observed that interference with Ccl19.1 function leads to a spectrum of dorsalized phenotypes at 27 hpf (Figure 6Ca,b) and altered expression of selected dorsal/ventral markers during blastula and gastrula stages (Figure 6Cc). The specificity of MO1-*ccl19.1* was confirmed with co-injection of synthetic *ccl19.1* RNA lacking the targeting sequence (Figure S5B). In addition, injections of a control MO harboring five mismatches in MO1-*ccl19.1* target sequence had no effect on embryo morphology (unpublished data) or expression of dorso-ventral markers at blastula stages (Figure S5C).

In support of Ccl19 and Ccr7 functioning as a ligand-receptor pair, embryos injected separately with low doses of MO1-*ccr7* or MO1-*ccl19.1* developed normally, whereas co-injection of the same doses of both MOs dorsalized embryos in a synergistic fashion (Figure 6D). Co-injections of *ccr7* and *ccl19.1* RNAs showed similar synergistic effects on ventralization (unpublished data). To ask whether the ventralizing activity of Ccl19.1 depended on normal Ccr7 function, we carried out epistasis experiments. Whereas *ccl19.1* RNA-injected embryos showed expanded *szl* expression (Figure 6Eb; 65%, *n* = 32), most embryos co-injected with MO1-*ccr7* showed little or no *szl* expression (Figure 6Ed; 84%, *n* = 38), as observed for *ccr7* morphants (Figures 1C and 6Ec; 92%, *n* = 13). Altogether, these data provide support for the notion that Ccl19.1 functions upstream of the Ccr7 GPCR to inhibit β-catenin and limit axis formation, likely as its specific ligand.

## Discussion

Since canonical Wnt, BMP, Nodal, FGF, and retinoic acid signaling pathways were shown to be involved in the specification and patterning of the embryonic axis in vertebrates over a decade ago [1],[66], no new signaling pathway has been implicated in this fundamental developmental process. Here, we uncover a previously uncharacterized role for the Ccr7 chemokine GPCR and its ligand, Ccl19.1, in zebrafish embryonic axis specification. A particularly intriguing finding is that Ccl19.1/Ccr7 signaling negatively regulates the Wnt/β-catenin pathway by a Gsk3β-independent mechanism. Importantly, our study reveals a novel layer of negative control of axis formation that does not involve a component of Wnt signaling. Moreover, it underscores the significance of the precise regulation of this first symmetry-breaking process during vertebrate embryogenesis.

We provided several lines of evidence that Ccr7 GPCR and Ccl19.1 chemokine are required and sufficient to negatively regulate β-catenin and axis formation (Figures 1, 2, and 6) in a Gsk3β-independent manner (Figure 3). Because MOs cannot inhibit Ccr7 and Ccl19.1 proteins that are likely maternally contributed, and there is a narrow time window for injected MOs to effectively interfere with translation of the maternally deposited *ccr7/ccl19.1* mRNAs, the incompletely penetrant and variable phenotypes described here likely represent a partial loss of *ccr7* and *ccl19.1* function. It is noteworthy that axis specification defects of variable penetrance and expressivity are observed in embryos homozygous for a nonsense mutation in the *boz* gene [41]. Additionally, the zebrafish genome encodes four homologs encoding putative Ccr7 ligands, Ccl19.1, Ccl19.2, Ccl19.3, and Ccl21/25 [65], from which three (Ccl19.1, Ccl19.2, and Ccl21/25) are maternally expressed (SW, JS, and LSK, unpublished data). All three predicted ligands are considered equally potent to activate Ccr7 signaling, based on their comparable overall similarity to the mammalian CCR7 ligands CCL19 and CCL21 [37], which can elicit different immune responses [67],[68]. Belonging to the class of chemokine GPCRs, CCR7 is thought to activate intracellular signaling only when bound to its cognate ligands [69], resembling the well-studied ligand-receptor pair CXCL12-CXCR4 [31],[32]. Thus, the Ccr7 overexpression phenotypes described here are most likely dependent on the presence of its ligands in the early embryo. Interestingly, and similarly to Ccr7, gain-of-function phenotypes upon overexpression in zebrafish embryos have been reported for several GPCRs, including Cxcr4 [31], and Agtrl1b [35],[36]. Further studies are needed to determine whether other putative Ccr7 ligands, in addition to Ccl19.1 or other GPCR signaling pathways, are involved in the regulation of axis formation. Moreover, given the dynamic expression pattern of *ccr7* and *ccl19.1* at later stages of embryogenesis (Figure S1B and unpublished data), it will be interesting to ask whether they have other developmental roles.

In mammals, Ccr7 GPCR is known to elicit intracellular Ca^2+^ signaling as one of its downstream responses [57]. In support of the notion of Ccr7 acting as a GPCR during axis formation, we demonstrated that misexpression of two dominant-negative forms of Ccr7, shown in mammalian cell culture to impair its interaction with G proteins, phenocopied the dorsalization observed in MO1-*ccr7* injected embryos (Figure 4A). Likewise, GDP-β-S suppressed the ventralizing effect of Ccr7 overexpression (Figure 4B). Further, we showed that during early cleavage stages, Ccr7 and/or Ccl19.1 overexpression increased frequencies of Ca^2+^ transients that occur homogenously in the superficial cells of early zebrafish blastula [20],[56], while depleting Ccr7 and/or Ccl19.1 had the opposite effect (Figures 4F and S4A,B). Our observations that treatments with thapsigargin and 2-APB, drugs that inhibit Ca^2+^ transients (Figures 4F and S4A,B), elevate β-catenin levels (Figure 4E) and promote expression of β-catenin-dependent organizer genes (Figure S4C) suggest that Ccr7 inhibits β-catenin and axis formation, via its GPCR activity, to promote intracellular Ca^2+^ transients.

**Figure 7:**
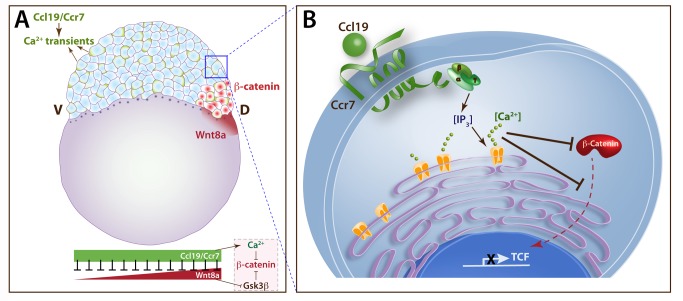
Model of maternal Ccr7 signaling during axis determination. (A) Whole-embryo view: maternal Ccl19/Ccr7 functions in early zebrafish blastula (depicted here as 256- to 512-cell stage) as a chemokine ligand/GPCR pair to promote Ca^2+^signaling (shown in green), exerting a tonic inhibition on the maternal Wnt/β-catenin activity in a Gsk3β-independent fashion, allowing nuclear accumulation of β-catenin only at the dorsal side (shown in red), where Wnt8 activity is high (shown in dark red). (B) Single-cell view: ligand binding (Ccl19 shown here) activates Ccr7, which induces Ca^2+^ release through heterotrimeric G proteins to inhibit the protein level of β-catenin and its nuclear localization, thus limiting the size of the dorsal blastula and later gastrula organizer.

The significance of Ca^2+^ signaling in axial patterning has been previously suggested [17],[18], and Ca^2+^ regulators have been implicated in embryonic axis formation, including NFAT [21] and Wnt5 [23]. Also consistent with the antagonism between Ca^2+^and the Wnt/β-catenin pathway during axis formation is the report of increased intracellular Ca^2+^ in the ventralized maternal-effect zebrafish mutant, *hecate* [70]. A number of studies using pharmacological inhibitors have implicated a signal transduction pathway dependent on the phosphatidylinositol (PI) cycle upstream of Ca^2+^ release from intracellular organelles in limiting dorsal axis formation [56],[58]. Moreover, three genes encoding IP_3_ receptors that mobilize intracellular Ca^2+^ stores upon activation by IP_3_ are expressed in zebrafish blastula [19], and IP_3_ concentration increases after 32-cell stage during axis specification [20]. Whereas GPCRs belong to a predominant class of cell surface receptors that activate the PI cycle, the identity of the putative GPCR(s) involved in embryonic axis formation remained elusive. Indeed, it was proposed that non-canonical Wnt signaling via Fz receptors, which share a seven-pass transmembrane structure with GPCRs, promotes Ca^2+^ release and limits axis formation in zebrafish [26],[27]. However, the requirement for non-canonical Wnts in limiting β-catenin and axis formation remains unclear [24],[25]. Here, we identify Ccr7 and Ccl19.1, a classic chemokine receptor-ligand pair, as suitable candidates for the hypothesized GPCR signaling pathway regulating axis formation because of their ability to promote Ca^2+^ transients, as well as to antagonize β-catenin and axis formation (see Figure 7). Based on the observations that induction of dorsal markers and increased β-catenin levels upon inhibition of Ca^2+^ by thapsigargin suppresses the ventralization caused by *ccr7* gain-of-function (Figures 4D), we propose that intracellular Ca^2+^ signaling downstream of Ccl19.1/Ccr7 is required to inhibit β-catenin and axis formation. However, the exact molecular mechanisms and involvement of signals downstream of Ccl19.1/Ccr7, in addition to Ca^2+^, remain to be investigated. Also, it will be important to determine whether and/or how Ccl19.1/Ccr7 interact with Wnt5/Fz [27], and/or possibly other GPCRs, in the negative regulation of β-catenin by Ca^2+^ during axis formation.

We propose a model in which Ccr7 chemokine GPCR and its Ccl19.1 ligand, ubiquitously expressed in the zebrafish zygote, promote intracellular Ca^2+^ transients to limit the maternal Wnt/β-catenin level and nuclear localization, and consequently the dorsal-specific gene network, to ensure a correct establishment of the embryonic axis in zebrafish (Figure 7). The formation of the Nieuwkoop center requires the microtubule-dependent transport of dorsal determinants that promote the nuclear localization of maternal β-catenin [71],[72]. Recent studies provided evidence for *wnt8a* mRNA being one such determinant that is transported during first cleavages from the vegetal pole towards one side of the blastoderm margin. This asymmetric dorsal transport of *wnt8a* mRNA, along with broad expression of Wnt antagonists in all blastomeres, is thought to create a dorsal to ventral gradient of Wnt8a/β-catenin activity [73]. Thus, only the Nieuwkoop center accumulates a sufficient amount of nuclear β-catenin to initiate the dorsal axis specification via overcoming the global antagonizing effect of the Ccl19.1/Ccr7/Ca^2+^ signaling, which limits the domain of the blastula organizer and prevents formation of ectopic organizers. A parallel for such a ubiquitous inhibitor regulating axis formation is the function of Lnx-2b E3 ubiquitin ligase, which limits the SMO domain by negatively regulating Boz protein stability in the zebrafish embryo [74]. Such a role for Ccl19.1/Ccr7 signaling is consistent with the prevalence of negative control mechanisms during early axis formation, given the dynamic nature of the organizing centers [4].

Surprisingly, *ich* mutants having reduced levels of β-catenin-2 [16] appear to be highly sensitive to modulation of Ccr7 and Ca^2+^ signaling. Whereas WT embryos injected with MO1-*ccr7* largely manifest enlarged SMO and only infrequently form ectopic organizers (Figure 1C), *ich/β-catenin2* mutants injected with MO1-*ccr7* frequently form partial double axes (Figure 2A). It is conceivable that in WT embryos, the Wnt8-dependent β-catenin gradient [73] inhibits formation of additional organizing centers, as is well established for the SMO during late blastula and gastrula stages [4]. Whereas, in *ich/β-catenin-2* mutants, such inhibitory activity is absent, allowing for the formation of ectopic organizers. Whether such negative interaction exists, and its molecular nature, remains to be determined. Alternatively, *ich* mutants have defects in addition to reduced levels of β-catenin protein.

One key question concerns the underlying molecular mechanism by which Ccr7-induced Ca^2+^ transients regulate Wnt/β-catenin activity. We provided several lines of evidence that the Ccl19.1/Ccr7/Ca^2+^ pathway inhibits posttranscriptionally the level and nuclear localization of the endogenous maternal β-catenin (Figure 2D and 2E) as well as exogenous ΔNβ-catenin that is resistant to Gsk3β-dependent proteasome degradation (Figure 3D and 3E). However, because Lactacystin partially suppressed Ccr7-dependent downregulation of β-catenin (Figure S3), Ccr7 signaling may reduce the level of β-catenin by promoting its degradation and indirectly inhibit its nuclear localization. It will be particularly important to determine whether Ccr7/calcium activates a negative regulator of β-catenin in a cell-autonomous manner or Ca^2+^ transients in superficial blastomeres stimulate a non-cell-autonomous signal that inhibits β-catenin. Several reports hint at potential mechanisms via which Ca^2+^ could inhibit β-catenin during axis formation. It has been shown in mammalian cell culture that the Wnt5/Ca^2+^ pathway antagonizes the canonical Wnt pathway by promoting Gsk3β-independent β-catenin degradation that is Siah2- and APC-dependent [75]. In another report, activated Gαq pathway inhibited β-catenin stimulated cell proliferation in SW480 cells via triggering a Ca^2+^-dependent nuclear export and a calpain-mediated degradation of β-catenin in the cytoplasm [76]. The nucleocytoplasmic shuttling of β-catenin is under precise control during embryogenesis, and multiple regulators, including Chibby [77] and JNK [78], have been implicated in this process. Moreover, Gαq has been shown to negatively regulate the Wnt/β-catenin pathway and dorsal development in *Xenopus* embryos [79]. Thus, it is tempting to speculate that, upon Ccl19.1 binding, activated Ccr7 receptors engage Gαq (or Gαi) and initiate the PI cycle to mobilize the intracellular Ca^2+^, which in turn results in the nuclear export of β-catenin and its subsequent degradation.

One important question is whether the negative regulation of β-catenin by Ccl19.1/Ccr7 described herein is conserved in other vertebrates during axis formation or other processes. Whereas the involvement of Wnt/β-catenin activity during axis formation appears to be conserved between frog, fish, and mouse, the specific ligands involved differ. For example, *wnt11* was proposed to positively regulate β-catenin during axis formation in *Xenopus* [80], whereas recent studies implicate *wnt8a* in the zebrafish axis formation [73], and the identity of Wnt ligand regulating axis formation in mammalian embryos remains elusive. Given the proposed involvement of Gαq in negative regulation of the Wnt/β-catenin pathway and dorsal development in *Xenopus* embryos [79], it is tempting to speculate that the role of chemokine GPCRs as negative regulators of β-catenin during embryonic axis formation is conserved among vertebrates, while the identity of specific receptors and ligands involved may differ between different species, with the murine Ccr7 being dispensable for this process [81].

Upregulated Wnt/β-catenin pathway often leads to overproliferative cells/tissues that eventually become cancerous, particularly in colorectal tumorigenesis [82]. The genetic interaction between Ccl19/Ccr7 and Wnt/β-catenin pathways we describe here may provide a new insight into the molecular basis of carcinogenesis. We speculate that a reduction of CCR7 (or its ligands, CCL19 and CCL21) expression in some tissues permits higher β-catenin activity that is oncogenic. Indeed, several recent studies of colorectal adenoma tissues report reduced RNA or protein expression levels of CCR7 or its ligands, compared to surrounding normal tissues [83]–[85]. Additionally, injecting synthetic human *CCR7* RNA ventralized zebrafish embryos (Figure S6), suggesting a conserved activity of β-catenin inhibition. Therefore, future studies in colorectal cancer tissues are warranted and may shed new light on the involvement of CCR7 signaling in colorectal tumorigenesis or other cancer types that depend on the Wnt/β-catenin pathway. CCL19/CCR7 would afford a new avenue to target Wnt/β-catenin signaling, given the highly drugable nature of chemokine receptors.

Given the prominent roles of the Wnt/β-catenin pathway in stem cell biology and embryonic stem (ES) cell differentiation [86], it is intriguing that Ccr7 is expressed in mouse ES cells, and its expression is downregulated during embryoid body differentiation [87]. Furthermore, the PGE2/Wnt interaction regulates stem cell development and tissue regeneration [88], while in dendritic cells, PGE2 acts as a potent inhibitor of CCL19 expression [89]. Hence, it is plausible that Ccl19/Ccr7, or more generally chemokine GPCR signaling, is also involved in embryonic stem cell differentiation.

In conclusion, our work delineates a novel, Gsk3β-independent negative control mechanism of β-catenin and implicates Ccr7 and its Ccl19.1 ligand as a long-hypothesized GPCR signaling pathway regulating vertebrate axis formation. Future studies are needed to elucidate the molecular interactions between Ccr7 and the known regulators of Wnt/β-catenin pathway in developmental processes, homeostasis, and disease.

## Materials and Methods

### Zebrafish Strains and Maintenance

Embryos were obtained from natural matings and staged according to morphology as described [90]. With the exception of *ich^p1^* (a gift from Dr. Gianfranco Bellipanni, Temple University) [7], all experiments were performed using wild-type (AB*) fish.

### Embryo Injection and Plasmid Construction

Zebrafish zygotes were injected with antisense morpholino oligonucleotides (MOs) or synthetic RNA at the one-cell stage within 10 min post-fertilization. The injected MOs included MO1-*ccr7* (5′-TTGCAGATGACTTTCTGATTGAACG-3′) and MO1-*ccl19.1* (5′-TCTGGAGAAGCTAGAAGAGTGTTGA-3′), control 5 mismatch MO-*ccr7* (5′-TTCCACATCACTTTGTCATTGAACG-3′), and control 5 mismatch MO-*ccl19.1* (5′-TCTGCACAAGCTACAAGACTCTTGA-3′). Synthetic RNA used included *β-catenin*, *ΔNβ-catenin-GFP* [52], *bozozok*/*dharma* (*boz*) [41],[47], *ccr7* (GenBank: BC142913.1), and *ccl19.1* (GenBank: BC122386.1). The C-terminal deletion of Ccr7 (aa 1–330; DN1-Ccr7) was generated by PCR using cDNA encoding full-length Ccr7 (aa 1–372) as a template. The Ccr7-DNY mutant (DN2-Ccr7) was generated by site-directed mutagenesis on aa 160 (R to N) using *pCS2-Ccr7* as a template [55]. All RNA constructs were cloned in the *pCS2* vector and synthesized with the in vitro transcription kit (mMESSAGE mMACHINE; Ambion).

### Pharmacological Treatments

Embryos were incubated in 0.3 M LiCl in embryo medium at the 128-cell stage for 10 min, then rinsed three times in 0.3× Danieau [54], and cultured until the desired stage. For Ca^2+^ inhibitors, embryos were treated with 4 µM thapsigargin (Sigma) from 32–64-cell-stage for 45 min or with 50 µM 2-APB (2-Aminoethyldiphenyl borinate; Sigma) from 256-cell-stage for 45 min and then washed three times with 0.3× Danieau, according to Westfall et al. [58] and Ma et al. [56], respectively. For Proteasome inhibition, the dechorionated embryos were incubated in 10 µM Lactacystin (Cayman) from 2-cell stage to 4 hpf [91]. Control embryos were treated with 0.1% DMSO. The GPCR inhibitor, GDP-β-S (Calbiochem), was injected at the stock concentration (20 µM) according to Ma et al. [56].

### Whole-Mount Immunohistochemistry and in situ Hybridization

Embryos were collected at the indicated stage and fixed overnight in 4% paraformaldehyde in PBS, permeabilized in 0.5% Triton in PBS for 30–60 min at room temperature, and labeled with anti-β-catenin mAb (1/250; Sigma T6557) overnight at 4°C. Whole-mount in situ hybridization (WISH) was performed as described [92]. Probes used are listed in Text S1.

### Western Blot Analysis

Embryos were manually deyolked in 0.3× Danieau solution and homogenized in RIPA buffer at 4 h post-fertilization (hpf) to extract proteins, which were then separated by SDS-PAGE and analyzed by western blotting. The following antibodies were used: mouse anti-β-catenin (Sigma C7207; 1/1,000), rabbit anti-GFP (Torrey Pines TP401; 1/1,000), mouse anti-actin (Sigma A5441; 1/2,000), anti-mouse IgG IRdye700DX conjugated (Rockland 610-730-124; 1/5,000), and anti-rabbit IgG IRdye800DX conjugated (Rockland 611-132-122; 1/5,000). The signal intensity was measured using Odyssey Infrared Imaging System.

### Quantitative RT-PCR (qRT-PCR)

RNA was isolated with Trizol (Invitrogen), followed by DNase treatment (Ambion). cDNA synthesis and qRT-PCR using SYBR green were performed according to the manufacturer's protocol (Biorad). Three samples at indicated stages were collected and reactions were performed at least twice on each sample to determine ΔC_T_ and translated into relative fold expression (estimation of 1.6^ΔC^_T_). Results are shown as relative fold ± SEM and subjected to Student's *t* test analysis to determine statistical significance (*p*<0.05). Primers used are listed in Text S1.

### Time-Lapse Ca^2+^ Imaging

Protocols were adapted from Freisinger et al. [93] and Ma et al. [56]. Zygotes were injected at the one-cell stage with 1–2 nL of the mixture of Calcium Green-1 (CaG) dextran and Tetramethyl-Rhodamine dextran (10 Kd, Invitrogen; 0.1% w/v, 0.01%, respectively, in 120 mM KCl, 20 mM HEPES pH 7.5) and maintained at 28.5°C in the dark. At the 128-cell stage (2.25 hpf), manually dechorionated embryos were examined with the Zeiss 510 META confocal system or the Quorum spinning disk confocal inverted microscope. The Rhodamine dextran was used to monitor changes in fluorescence that occur independently of changes in Ca^2+^ concentration. A timed series of images (one image per 8–10 s), focusing on the superficial layer, was collected for 20 min, initiating between the 256- to 512-cell stages (2.5 to 2.75 hpf). Transient Ca^2+^ release peaks were analyzed frame-by-frame from Volocity software (Perkin Elmer) ratiometric images. Alternatively, changes in brightness were quantified from raw CaG and Rhodamine images separately. In Excel, we calculated the change in CaG intensity [average pixel intensity CaG (time2/time1)] normalized to the change in the Ca^2+^ insensitive channel [average pixel intensity Rhodamine (time2/time1)]. Cells manifesting a 5% or greater change were counted as calcium transients. Ratiometric images were made using Metamorph “Ratio Images” function (Molecular Devices).

## Supporting Information

Figure S1 Zebrafish *ccr7* gene sequence and expression, MO1*-ccr7* efficiency, and specificity tests and expression of region-specific markers in *ccr7* morphants. (A) Multiple sequence alignments of selected vertebrate Ccr7 proteins. Mm, *Mus musculus*; Dr, *Danio rerio*; Xl, *Xenopus laevis*; Hs, *Homo sapiens*. Alignments were carried out using the MultAlin web-based software. (B) Spatiotemporal expression pattern of *ccr7* revealed by WISH. *ccr7* is expressed maternally (8-cell stage, 1.25 hpf) (a), and its transcripts are uniformly distributed until sphere stage (4 hpf) (b). At dome stage (4.5 hpf), a slight asymmetry in *ccr7* expression is observed (c). At shield stage, *ccr7* RNA is enriched dorsally (d–e). Lateral views, animal to the top (a, b, d), animal views (c, e). Scale bars in all panels, 200 µm. (C) Embryos injected with MO1-*ccr7* (20 ng) exhibited at 11 hpf elongated shape typical of dorsalization; penetrance is shown in the bottom panel. (D) Fluorescent image of zebrafish blastulae at 5.5 hpf injected at 1-cell stage with synthetic RNA encoding *ccr7 5′UTR-egfp* (a) and co-injected with 10 ng of MO1*-ccr7* (b). MO1*-ccr7* inhibited EGFP expression (*n* = 24/24) (b). Lateral views. (E) Expression of *szl* in control (a), MO1-*ccr7*-injected (c, 10 ng; *szl* expression reduced in 69%, *n* = 35), and MO1-*ccr7* and *ccr7* RNA (100 pg) co-injected embryos (c, *szl* expression reduced in 26%, *n* = 50). (F) Expression of dorsal and ventral markers in control uninjected embryos (a–h) and embryos injected with five base mis-matched control morpholino for *ccr7* (5-mm *ccr7* MO, 20 ng) (a′–h′) revealed by WISH: a, *n* = 20/20; a′, *n* = 22/22; b, *n* = 15/21; b′, *n* = 14/21; c, *n* = 22/22; c′, *n* = 21/21; d, *n* = 20/20; d′, *n* = 15/15; e, *n* = 18/18; e′, *n* = 17/17; f, *n* = 19/19; f′, *n* = 18/18; g, *n* = 19/19; g′, *n* = 16/16; h, *n* = 19/19; h′, *n* = 16/16. Animal views with dorsal to the right, when the dorsal side is recognizable. (G) Expression of dorsal and ventral markers in control uninjected embryos (a, b, c) and *ccr7* morphants (a′, c′, d′) revealed by WISH. Note expanded expression of *sqt* at 4 hpf (a, a′, 83%, *n* = 12); *hhex* at 6 hpf (b, b′, 80%, *n* = 20), and reduced expression of *vent* a 4.7 hpf (c, c′, 71%, *n* = 14). Animal views with dorsal to the right. (d) Quantification of the relative expression levels of *boz*, *mkp3*, *fgf3*, and *bmp2b* at 3.3 and 4 hpf. See text for details. * *p*<0.05 (Student's *t*-test). (H) Expression of dorsal and ventral markers in control uninjected embryos (a–h) and embryos injected with *ccr7* RNA (200 pg) (a′–h′) revealed by WISH: a, *n* = 20/20; a′, *n* = 10/16; b, *n* = 15/21; b′, *n* = 12/15; c, *n* = 22/22; c′, *n* = 10/14; d, *n* = 20/20; d′, *n* = 10/15; e, *n* = 18/18; e′, *n* = 12/15; f, *n* = 19/19; f′, *n* = 9/13; g, *n* = 19/19; g′, *n* = 9/13; h, *n* = 19/19; h′, *n* = 9/12. Animal views with dorsal to the right, when the dorsal side is recognizable. Arrowheads mark the expansion of ventral expression domain of *vox* (e and e′).
(TIF)

Figure S2 Ccr7 acts upstream of Boz and proximally to β-catenin. (A) The dorsalized phenotypes caused by Boz overexpression (50 pg; *n* = 15/15) (b) compared to control embryos (a), could not be suppressed by Ccr7 overexpression (150 pg; *n* = 16/16) (c). Lateral views of embryos at 30 hpf. (B) The expansion of *gsc* expression induced by injection of *boz* RNA (b, 50 pg; *n* = 20/20), compared to control (a), remained unchanged in embryos co-injected with *ccr7* RNA (c, 150 pg, *n* = 18/18). (C) Penetrance of the strongly ventralized *ich* mutant phenotype (a; 100%, *n* = 25) was reduced by injection of synthetic RNA encoding β-catenin (b; 50 pg, 6%, *n* = 15). This rescue was inhibited by co-injection of *ccr7* RNA (c, c′, 150 pg, strongly ventralized phenotype 81%, *n* = 21). Lateral views at 27 hpf. (D) Co-injection of MO1-*ccr7* enhanced the penetrance and expressivity of the dorsalized phenotypes of WT 27 hpf embryos injected with RNA encoding ΔN-β-catenin. (E) Ccr7 gain-of-function decreased both levels of endogenous β-catenin and ectopic β-catenin-GFP. Western blotting of β-catenin and GFP protein from uninjected control, *β-catenin-GFP* RNA (10 pg) injected, or *β-catenin-GFP* RNA (10 pg)/*ccr7* RNA (200 pg) co-injected embryos at 4 hpf. Graphs below show the relative protein level (signal intensity) quantified from three separate immunoblots. * *p*<0.05.
(TIF)

Figure S3 Effect of Lactacystin on Gsk3β and Ccr7-dependent β-catenin downregulation. Confocal microscope images of 4 hpf stage embryos injected with β-catenin-GFP RNA (10 pg) (a–c′) that were injected with RNA encoding Gsk3β (200 pg) (b, b′) or Ccr7 (200 pg) (c, c′) and treated with Lactacystin (a′, b′, c′). Animal views.
(TIF)

Figure S4 Effects of *ccr7* and thapsigargin on Ca^2+^ transients in superficial blastomeres. (A) Examples of Ca^2+^ transients at about 256-cell stage in ratiometric images (minimum calcium ratio is 0, maximum is 10). (a) In WT embryo, arrowheads point out increased Ca^2+^ level at near time points (still images). Note the rapid changes of Ca^2+^ peaks (compare a to b, which is 35 s later). (c) The average pixel intensity for the Ca^2+^ sensitive dye, Calcium Green-1 dextran (green) is shown for the cells (numbered arrowheads) and for the Ca^2+^ insensitive Tetramethyl Rhodamine dextran (red and black) over a 400 s/50 frame time period. (d, e, f) In MO1-*ccr7*/MO1-*ccl19.1-*injected embryos, one cell exhibits a Ca^2+^ transient at 35 s interval. (g, h, i) In *ccr7*/*ccl19.1* RNA-injected embryo, one cell showed Ca^2+^ transient over 30 s interval. (j, k, l) In thapsigargin-treated embryos no Ca^2+^ transients were observed over 400 s interval. (B) Number of Ca^2+^ transients normalized to mean for WT. Injection of MO1-*ccr7* and *ccr7* RNA were normalized to WT1 group. Injection of MO1-*ccr7*/MO1-*ccl19.1* and *ccr7*/*ccl19.1* RNA were normalized to WT2 group. * *p*<0.05. (C) *mkp3* expression at 4 hpf in WT embryos (a) treated with 4 µM thapsigargin (b) and 50 µM 2-APB (c). (D) Images of control embryos (a) and embryos treated at cleavage stages with thapsigargin (b) or 2APB (c) at 30 hpf.
(TIF)

Figure S5 Ccl19.1 overexpression and test of MO1*-ccl19.1* specificity. (A) Dose-dependent ventralization of WT embryos injected with *ccl19.1* RNA (100–300 pg). V1–V3 classes are defined as in Figure 1B. (B) Ccl19.1 morphants exhibited at 11 hpf dorsalized elongated shape (b), which was suppressed by co-injection of *ccl19.1* RNA lacking the MO1-*ccl19.1* target site (c, d). (C) Expression of dorsal and ventral markers in control uninjected embryos (a–h) and embryos injected with a five base mis-matched control morpholino for *ccl19.1* (5-mm *ccl19.1* MO, 4 ng) (a′–h′) revealed by WISH: a, *n* = 20/20; a′, *n* = 16/16; b, *n* = 15/21; b′, *n* = 9/14; c, *n* = 22/22; c′, *n* = 17/17; d, *n* = 20/20; d′, *n* = 15/16; e, *n* = 18/18; e′, *n* = 19/19; f, *n* = 19/19; f′, *n* = 15/15; g, *n* = 19/19; g′, *n* = 15/16; h, *n* = 19/19; h′, *n* = 15/15. Animal views with dorsal to the right, when the dorsal side is recognizable. (D) Expression of dorsal and ventral markers in control uninjected embryos (a–h) and embryos injected with *ccl19.1* RNA (200 pg) (a′–h′) revealed by WISH: a, *n* = 20/20; a′, *n* = 10/14; b, *n* = 15/21; b′, *n* = 10/14; c, *n* = 22/22; c′, *n* = 10/14; d, *n* = 20/20; d′, *n* = 9/17; e, *n* = 18/18; e′, *n* = 13/17; f, *n* = 19/19; f′, *n* = 9/15; g, *n* = 19/19; g′, *n* = 12/15; h, *n* = 19/19; h′, *n* = 9/14. Animal views with dorsal to the right, when the dorsal side is recognizable.
(TIF)

Figure S6 Overexpression of human CCR7 phenocopies ventralization caused by zebrafish Ccr7 gain-of-function. (A) Morphology of control (a) and human *CCR7* RNA-injected (200 pg) embryos (b, b′; ventralized phenotype seen in *n* = 8/25). Lateral views with anterior to the left. (B) Expression of *szl* in control and human CCR7 overexpressing gastrulae at shield stage, 6 hpf (200 pg, reduced expression seen in *n* = 10/15). Animal views with dorsal to the right.
(TIF)

Movie S1 An example of time-lapse calcium imaging of a control embryo. Actual time frame, 5 min.
(MOV)

Movie S2 An example of time-lapse calcium imaging of an embryo overexpressing Ccr7 RNA. Actual time frame, 8 min.
(MOV)

Movie S3 An example of time-lapse calcium imaging of an embryo injected with MO1-*ccr7* (10 ng). Actual time frame, 4 min.
(MOV)

Text S1 qRT-PCR primer sequences and in situ probes used in this study.
(DOC)

## Abbreviations

AP: anteroposterior
DV: dorsoventral
ES cell: embryonic stem cell
GPCR: G-protein coupled receptor
hpf: hours post-fertilization
MO: morpholino oligonucleotide
PI: phosphatidylinositol
SMO: Spemann-Mangold organizer
WISH: whole mount in situ hybridization
WT: wild type
