# Supplementary figures and images for: Chemokine GPCR Signaling Inhibits β-Catenin during Zebrafish Axis Formation
Source: PLoS Biol. 2012 Oct 9;10(10):e1001403. doi: 10.1371/journal.pbio.1001403 (PMC3467228; doi:10.1371/journal.pbio.1001403)

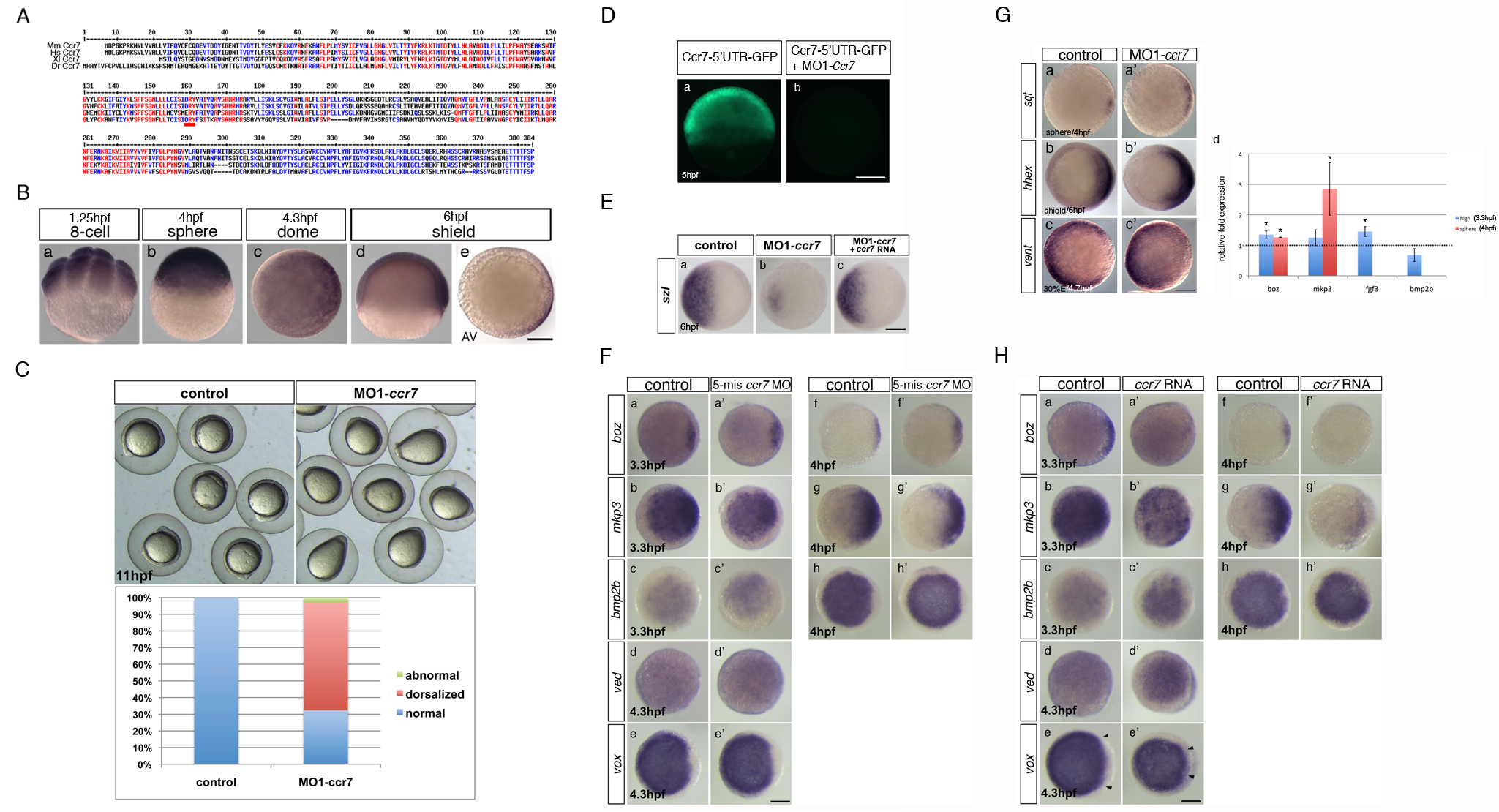

Supplement: Figure S1 — Zebrafish ccr7 gene sequence and expression, MO1-ccr7 efficiency, and specificity tests and expression of region-specific markers in ccr7 morphants. (A) Multiple sequence alignments of selected vertebrate Ccr7 proteins. Mm, Mus musculus; Dr, Danio rerio; Xl, Xenopus laevis; Hs, Homo sapiens. Alignments were carried out using the MultAlin web-based software. (B) Spatiotemporal expression pattern of ccr7 revealed by WISH. ccr7 is expressed maternally (8-cell stage, 1.25 hpf) (a), and its transcripts are uniformly distributed until sphere stage (4 hpf) (b). At dome stage (4.5 hpf), a slight asymmetry in ccr7 expression is observed (c). At shield stage, ccr7 RNA is enriched dorsally (d–e). Lateral views, animal to the top (a, b, d), animal views (c, e). Scale bars in all panels, 200 µm. (C) Embryos injected with MO1-ccr7 (20 ng) exhibited at 11 hpf elongated shape typical of dorsalization; penetrance is shown in the bottom panel. (D) Fluorescent image of zebrafish blastulae at 5.5 hpf injected at 1-cell stage with synthetic RNA encoding ccr7 5′UTR-egfp (a) and co-injected with 10 ng of MO1-ccr7 (b). MO1-ccr7 inhibited EGFP expression (n = 24/24) (b). Lateral views. (E) Expression of szl in control (a), MO1-ccr7-injected (c, 10 ng; szl expression reduced in 69%, n = 35), and MO1-ccr7 and ccr7 RNA (100 pg) co-injected embryos (c, szl expression reduced in 26%, n = 50). (F) Expression of dorsal and ventral markers in control uninjected embryos (a–h) and embryos injected with five base mis-matched control morpholino for ccr7 (5-mm ccr7 MO, 20 ng) (a′–h′) revealed by WISH: a, n = 20/20; a′, n = 22/22; b, n = 15/21; b′, n = 14/21; c, n = 22/22; c′, n = 21/21; d, n = 20/20; d′, n = 15/15; e, n = 18/18; e′, n = 17/17; f, n = 19/19; f′, n = 18/18; g, n = 19/19; g′, n = 16/16; h, n = 19/19; h′, n = 16/16. Animal views with dorsal to the right, when the dorsal side is recognizable. (G) Expression of dorsal and ventral markers in control uninjected embryos (a, b, c) and ccr7 morpha [file pbio.1001403.s001.tif]

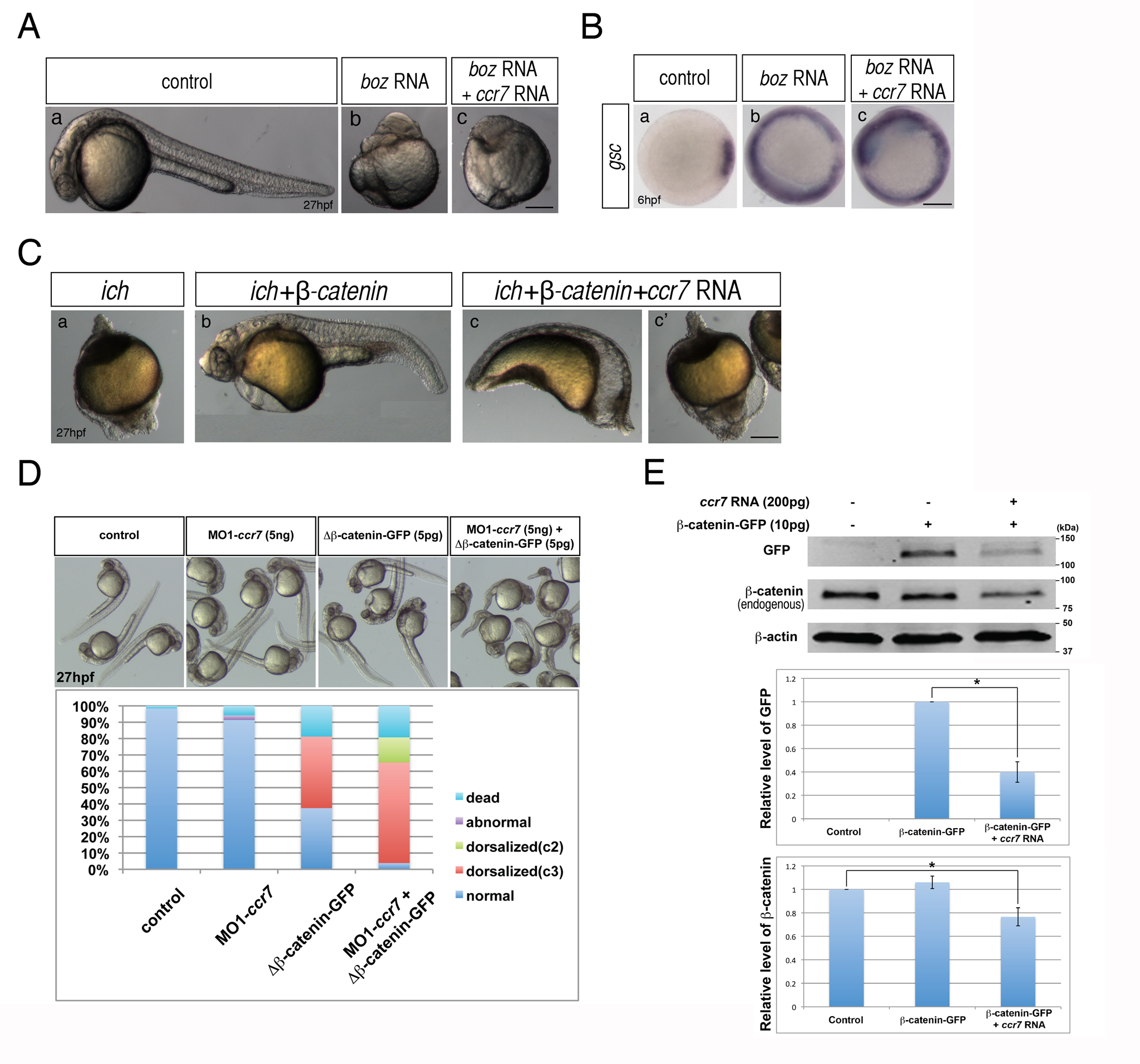

Supplement: Figure S2 — Ccr7 acts upstream of Boz and proximally to β-catenin. (A) The dorsalized phenotypes caused by Boz overexpression (50 pg; n = 15/15) (b) compared to control embryos (a), could not be suppressed by Ccr7 overexpression (150 pg; n = 16/16) (c). Lateral views of embryos at 30 hpf. (B) The expansion of gsc expression induced by injection of boz RNA (b, 50 pg; n = 20/20), compared to control (a), remained unchanged in embryos co-injected with ccr7 RNA (c, 150 pg, n = 18/18). (C) Penetrance of the strongly ventralized ich mutant phenotype (a; 100%, n = 25) was reduced by injection of synthetic RNA encoding β-catenin (b; 50 pg, 6%, n = 15). This rescue was inhibited by co-injection of ccr7 RNA (c, c′, 150 pg, strongly ventralized phenotype 81%, n = 21). Lateral views at 27 hpf. (D) Co-injection of MO1-ccr7 enhanced the penetrance and expressivity of the dorsalized phenotypes of WT 27 hpf embryos injected with RNA encoding ΔN-β-catenin. (E) Ccr7 gain-of-function decreased both levels of endogenous β-catenin and ectopic β-catenin-GFP. Western blotting of β-catenin and GFP protein from uninjected control, β-catenin-GFP RNA (10 pg) injected, or β-catenin-GFP RNA (10 pg)/ccr7 RNA (200 pg) co-injected embryos at 4 hpf. Graphs below show the relative protein level (signal intensity) quantified from three separate immunoblots. * p<0.05. (TIF) [file pbio.1001403.s002.tif]

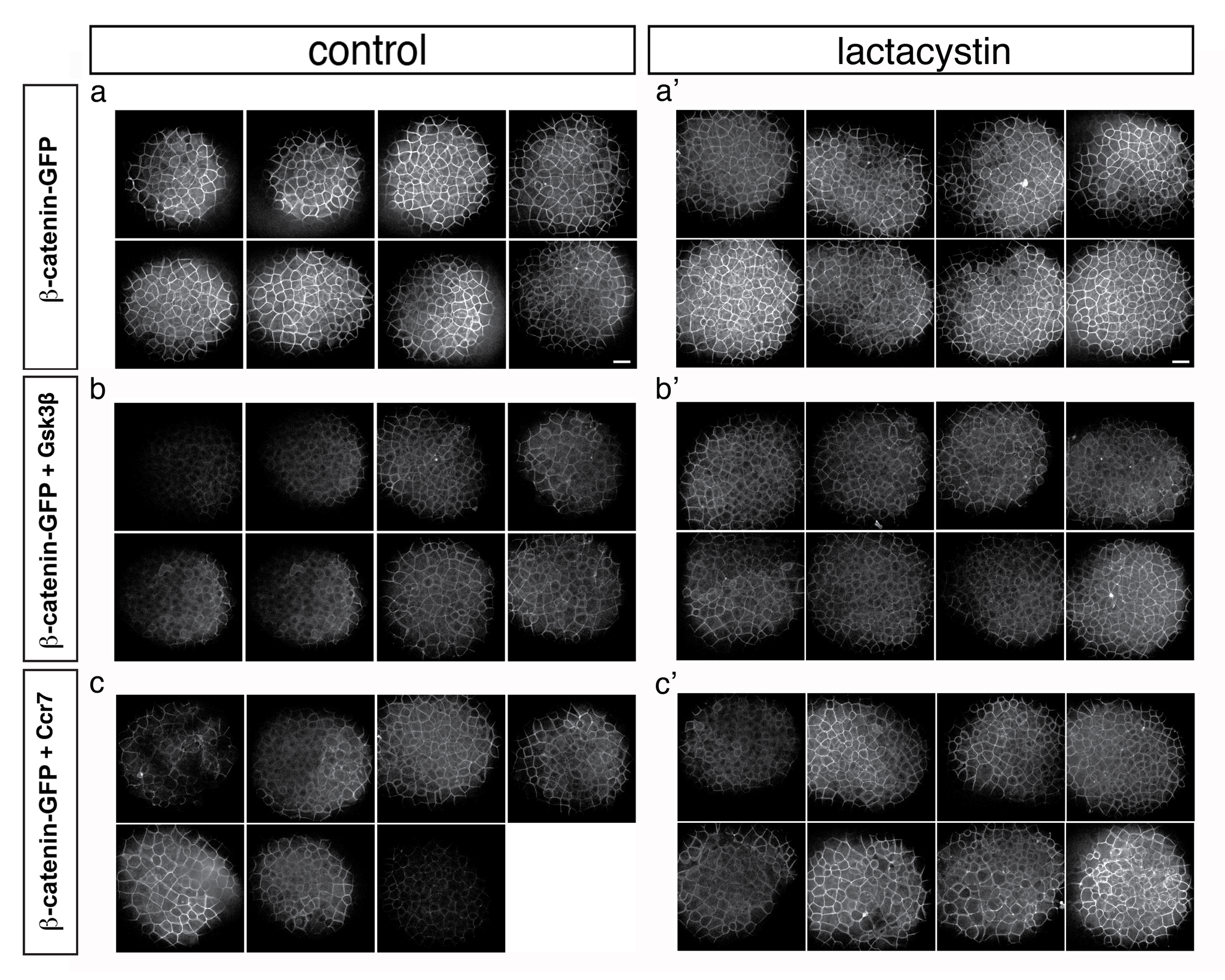

Supplement: Figure S3 — Effect of Lactacystin on Gsk3β and Ccr7-dependent β-catenin downregulation. Confocal microscope images of 4 hpf stage embryos injected with β-catenin-GFP RNA (10 pg) (a–c′) that were injected with RNA encoding Gsk3β (200 pg) (b, b′) or Ccr7 (200 pg) (c, c′) and treated with Lactacystin (a′, b′, c′). Animal views. (TIF) [file pbio.1001403.s003.tif]

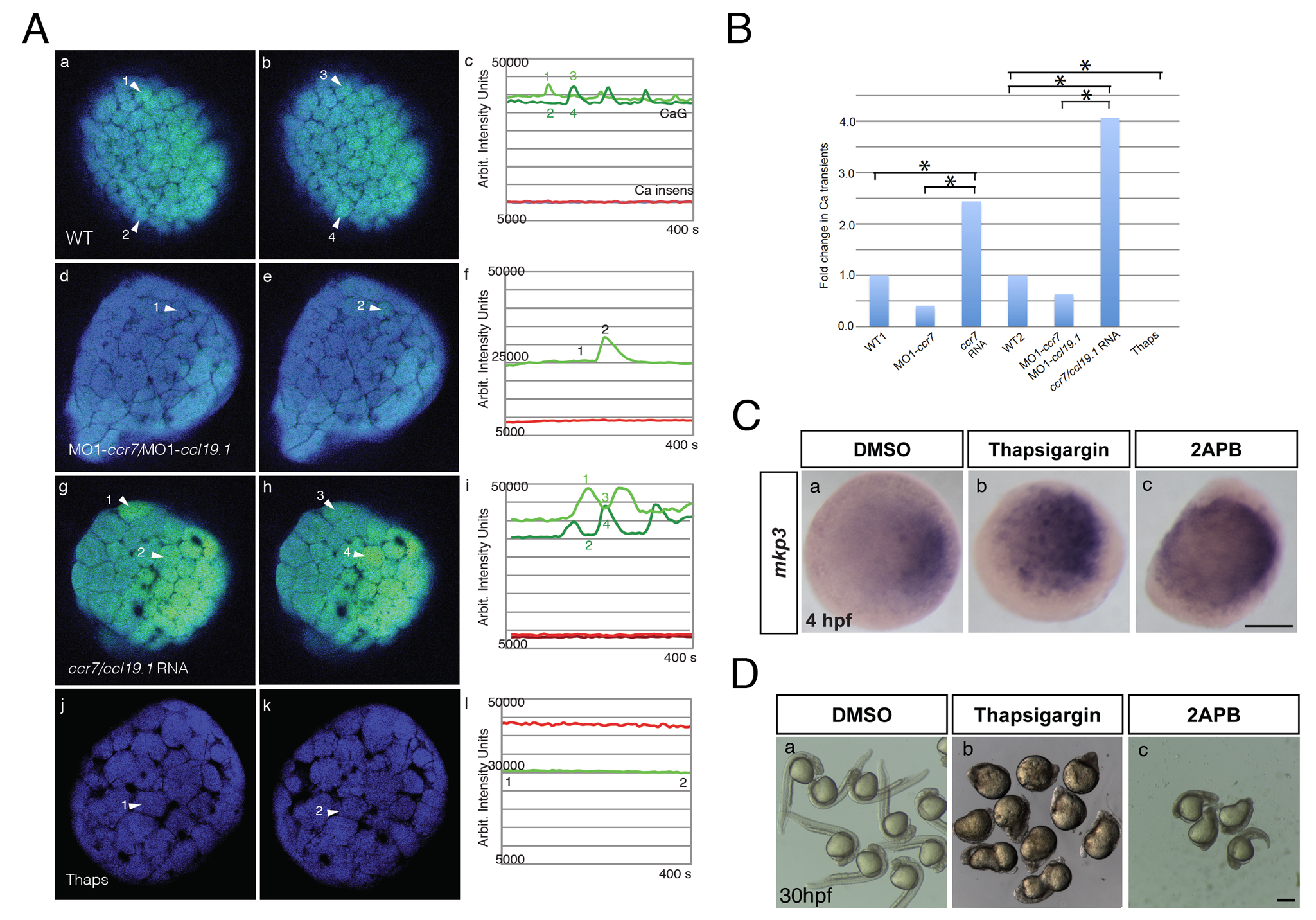

Supplement: Figure S4 — Effects of ccr7 and thapsigargin on Ca2+ transients in superficial blastomeres. (A) Examples of Ca2+ transients at about 256-cell stage in ratiometric images (minimum calcium ratio is 0, maximum is 10). (a) In WT embryo, arrowheads point out increased Ca2+ level at near time points (still images). Note the rapid changes of Ca2+ peaks (compare a to b, which is 35 s later). (c) The average pixel intensity for the Ca2+ sensitive dye, Calcium Green-1 dextran (green) is shown for the cells (numbered arrowheads) and for the Ca2+ insensitive Tetramethyl Rhodamine dextran (red and black) over a 400 s/50 frame time period. (d, e, f) In MO1-ccr7/MO1-ccl19.1-injected embryos, one cell exhibits a Ca2+ transient at 35 s interval. (g, h, i) In ccr7/ccl19.1 RNA-injected embryo, one cell showed Ca2+ transient over 30 s interval. (j, k, l) In thapsigargin-treated embryos no Ca2+ transients were observed over 400 s interval. (B) Number of Ca2+ transients normalized to mean for WT. Injection of MO1-ccr7 and ccr7 RNA were normalized to WT1 group. Injection of MO1-ccr7/MO1-ccl19.1 and ccr7/ccl19.1 RNA were normalized to WT2 group. * p<0.05. (C) mkp3 expression at 4 hpf in WT embryos (a) treated with 4 µM thapsigargin (b) and 50 µM 2-APB (c). (D) Images of control embryos (a) and embryos treated at cleavage stages with thapsigargin (b) or 2APB (c) at 30 hpf. (TIF) [file pbio.1001403.s004.tif]

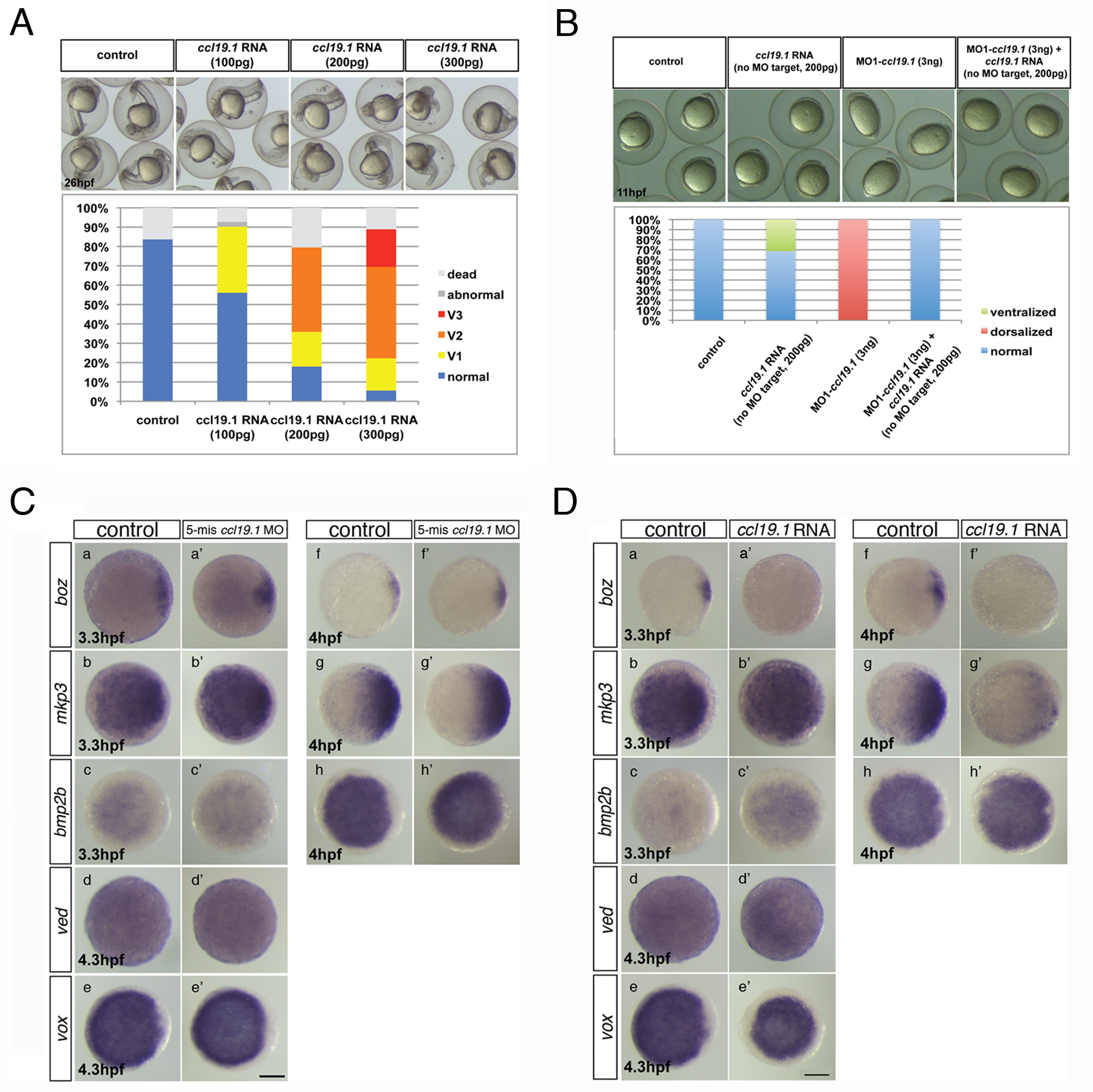

Supplement: Figure S5 — Ccl19.1 overexpression and test of MO1-ccl19.1 specificity. (A) Dose-dependent ventralization of WT embryos injected with ccl19.1 RNA (100–300 pg). V1–V3 classes are defined as in Figure 1B. (B) Ccl19.1 morphants exhibited at 11 hpf dorsalized elongated shape (b), which was suppressed by co-injection of ccl19.1 RNA lacking the MO1-ccl19.1 target site (c, d). (C) Expression of dorsal and ventral markers in control uninjected embryos (a–h) and embryos injected with a five base mis-matched control morpholino for ccl19.1 (5-mm ccl19.1 MO, 4 ng) (a′–h′) revealed by WISH: a, n = 20/20; a′, n = 16/16; b, n = 15/21; b′, n = 9/14; c, n = 22/22; c′, n = 17/17; d, n = 20/20; d′, n = 15/16; e, n = 18/18; e′, n = 19/19; f, n = 19/19; f′, n = 15/15; g, n = 19/19; g′, n = 15/16; h, n = 19/19; h′, n = 15/15. Animal views with dorsal to the right, when the dorsal side is recognizable. (D) Expression of dorsal and ventral markers in control uninjected embryos (a–h) and embryos injected with ccl19.1 RNA (200 pg) (a′–h′) revealed by WISH: a, n = 20/20; a′, n = 10/14; b, n = 15/21; b′, n = 10/14; c, n = 22/22; c′, n = 10/14; d, n = 20/20; d′, n = 9/17; e, n = 18/18; e′, n = 13/17; f, n = 19/19; f′, n = 9/15; g, n = 19/19; g′, n = 12/15; h, n = 19/19; h′, n = 9/14. Animal views with dorsal to the right, when the dorsal side is recognizable. (TIF) [file pbio.1001403.s005.tif]

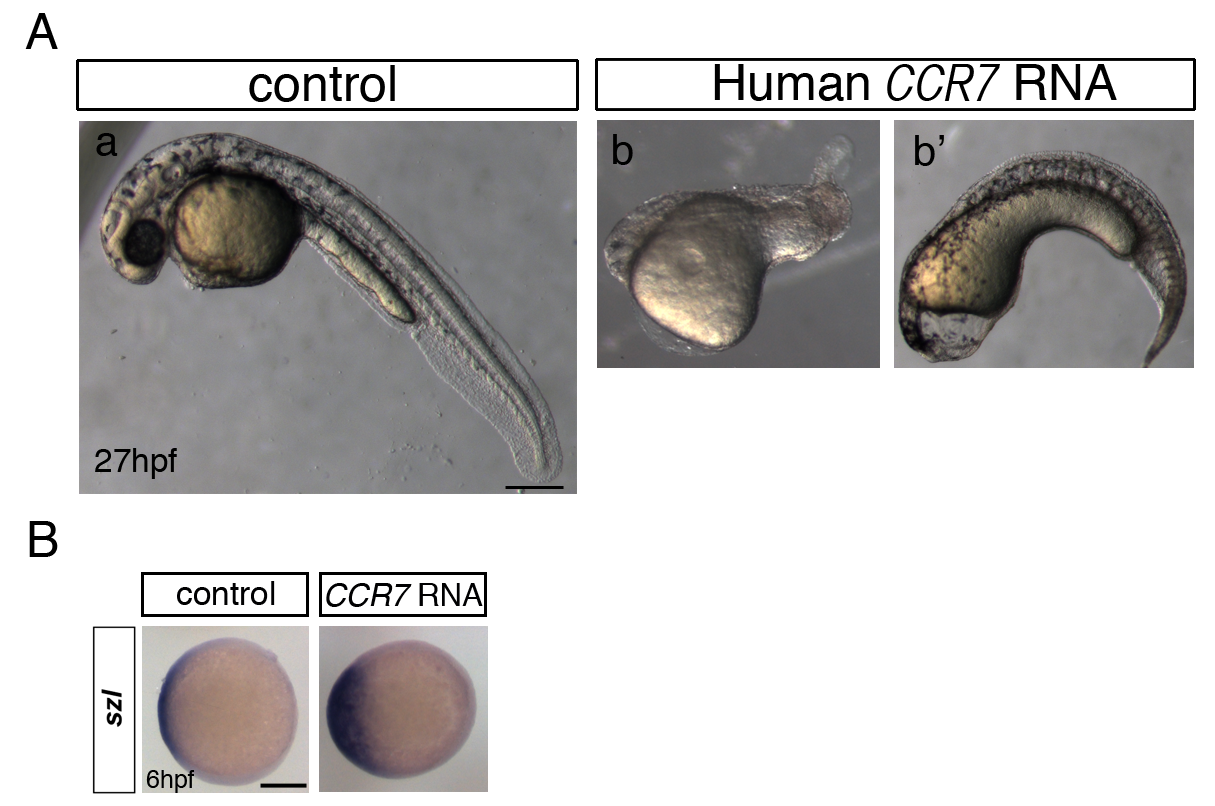

Supplement: Figure S6 — Overexpression of human CCR7 phenocopies ventralization caused by zebrafish Ccr7 gain-of-function. (A) Morphology of control (a) and human CCR7 RNA-injected (200 pg) embryos (b, b′; ventralized phenotype seen in n = 8/25). Lateral views with anterior to the left. (B) Expression of szl in control and human CCR7 overexpressing gastrulae at shield stage, 6 hpf (200 pg, reduced expression seen in n = 10/15). Animal views with dorsal to the right. (TIF) [file pbio.1001403.s006.tif]
