# Supplementary material for: Chemokine GPCR Signaling Inhibits β-Catenin during Zebrafish Axis Formation
Source: PLoS Biol. 2012 Oct 9;10(10):e1001403. doi: 10.1371/journal.pbio.1001403 (PMC3467228; doi:10.1371/journal.pbio.1001403)
Supplement: Text S1 — qRT-PCR primer sequences and in situ probes used in this study. (DOC) [file pbio.1001403.s010.doc]

**Text S1.**

**1. Primer sequences used in qRT-PCR experiments:**

*boz*-F, 5’-ccgtagccggttgtgaaacagc-3’

*boz*-R, 5’-ttcttgaaccacacccgcacag-3’

*mkp3*-F, 5’-cgttcagaggggttgtccg-3’

*mkp3*-R, 5’-cttccctgaacaggagaccc-3’

*fgf3*-F, 5’-GTGGCAATCAAGGGACTGTT-3’

*fgf3*-R, 5’-GCCGTGATGCATAAGTGTTG-3’

*bmp2b*-F, 5’-ACTGTCCAGCCTGAAAGGAA-3’

*bmp2b*-R, 5’-TGTGGAAGCCACTCGTACTG-3’

*gapdh*-F, 5’-gatacacggagcaccaggtt-3’

*gapdh*-R, 5’-gccatcaggtcacatacacg-3’

**2. In situ probes used and their respective references:**

***boz/dharma*** (Fekany et al., 1999; Yamanaka et al., 1998); ***dusp6/mkp3*** (Tsang et al., 2004); ***chd*** (Miller-Bertoglio et al., 1997); ***gsc*** (Schulte-Merker et al.,1994); ***bmp2b*** (Kishimoto et al., 1997); ***bmp4*** (Martinez-Barberá et al., 1997); ***ved*** (Shimizu et al., 2002); ***vent*** (Kawahara et al., 2000a); ***vox*** (Kawahara et al., 2000b); ***szl*** (Yabe et al., 2003); ***eve1*** (Joly et al., 1993); ***ndr1***/***sqt*** (Erter et al., 1998); ***hhex*** (Ho et al., 1999); ***myod1*** (Weinberg et al., 1996),

**Supplemental References**

**Erter CE, Solnica-Krezel L, Wright CVE (1998) Zebrafish nodal-related 2 encodes an early mesendodermal inducer signaling from the extraembryonic yolk syncytial layer. Dev Biol 204: 361-372.**

**Ho CY, Houart C, Wilson SW, Stainier DY (1999) A role for the extraembryonic yolk syncytial layer in patterning the zebrafish embryo suggested by properties of the hex gene. Curr Biol 9: 1131-1134.**

**Joly JS, Joly C, Schulte-Merker S, Boulekbache H, Condamine H. (1993) The ventral and posterior expression of the zebrafish homeobox gene eve1 is perturbed in dorsalized and mutant embryos. Development 119: 1261–1275.**

**Kawahara A, Wilm T, Solnica-Krezel L, Dawid IB (2000a) Functional interaction of vega2 and goosecoid homeobox genes in zebrafish. Genesis 28: 58-67.**

**Kawahara A, Wilm T, Solnica-Krezel L, Dawid IB (2000b) Antagonistic role of vega1 and bozozok/dharma homeobox genes in organizer formation. Proc Natl Acad Sci U S A 97: 12121-12126.**
